# Exploring antibiotic resistance in diverse homologs of the dihydrofolate reductase protein family through broad mutational scanning

**DOI:** 10.1126/sciadv.adw9178

**Published:** 2025-08-13

**Authors:** Karl J. Romanowicz, Carmen Resnick, Samuel R. Hinton, Calin Plesa

**Affiliations:** Department of Bioengineering, Phil and Penny Knight Campus for Accelerating Scientific Impact, University of Oregon, 1505 Franklin Boulevard, Eugene, OR 97403, USA.

## Abstract

Antimicrobial resistance studies often focus on individual protein variants, neglecting broader protein family dynamics. Dihydrofolate reductase (DHFR), a key antibiotic target, has been extensively studied using deep mutational scanning, yet resistance mechanisms across this diverse protein family remain poorly understood. Here, we developed a synthetic metagenomics approach using DropSynth, a scalable gene synthesis platform, to construct a phylogenetically diverse library of 1536 DHFR homologs. These sequences, primarily derived from host-associated metagenomes, represent 759 bacterial species, including many clinically relevant pathogens. A multiplexed in vivo assay tested their ability to restore metabolic function and confer trimethoprim resistance in an *Escherichia coli* ∆*folA* strain. Half of the synthetic homologs rescued the phenotype without supplementation, and mutant variants with up to five amino acid substitutions increased rescue rates to 90%, highlighting DHFR’s evolutionary resilience. Broad mutational scanning of DHFR homologs and 100,000 mutants revealed key insights into fitness and resistance, offering the most comprehensive analysis of complementation and inhibitor tolerance to date.

## Teaser

DropSynth technology enables scalable and cost-effective exploration of antibiotic resistance across the DHFR protein family.

## INTRODUCTION

Antimicrobial resistance is a pressing challenge in modern medicine, responsible for 4.95 million global deaths in 2019, surpassing the combined annual deaths from tuberculosis, malaria, and HIV/AIDS (*1*, *2*). The extensive use of antibiotics in clinical and industrial settings has fueled the emergence of bacterial resistance mechanisms, jeopardizing the treatment of common infections (*3*). Among antibiotic classes, dihydrofolate reductase (DHFR) inhibitors remain crucial for combating bacterial infections (*4*). DHFR, a key enzyme in folate biosynthesis, reduces dihydrofolate to tetrahydrofolate, essential for DNA synthesis and cell proliferation (*5*). Trimethoprim (TMP), a synthetic DHFR inhibitor, disrupts folate metabolism and inhibits bacterial growth (*6*). However, increasing occurrences of bacterial resistance to TMP underscores the need for continued research into its effectiveness across diverse members of the DHFR protein family.

The DHFR protein family, essential for DNA synthesis across all organisms, has undergone substantial divergent evolution, resulting in structural and functional variations with as little as 30% sequence identity between bacterial species (*7*). This diversity complicates efforts to address antibiotic resistance, as it enables bacteria to develop various strategies to evade DHFR inhibition. These strategies include mutations in the DHFR gene that alter the enzyme’s structure and reduce drug binding affinity (*8*), acquisition of plasmid-encoded DHFR genes that produce enzymes with lower drug sensitivity (*9*, *10*), overexpression of DHFR to counteract the inhibitory effects of TMP through increased enzyme abundance (*11*), and the development of alternative metabolic pathways that bypass DHFR activity (*12*, *13*). The diversity of these resistance mechanisms highlights the adaptability of bacteria and the challenges in sustaining the efficacy of DHFR inhibitors such as TMP as antibiotic treatments.

Given the critical role of DHFR in bacterial metabolism and the growing threat of antibiotic resistance, there is an urgent need to explore the sequence diversity within the DHFR family to understand TMP efficacy and develop strategies to counteract resistance. Conventional methods such as deep mutational scanning (DMS) have been invaluable for revealing protein function and evolution by systematically examining how mutations affect functional landscapes, evolutionary constraints, and structure-function relationships (*14*–*16*). However, DMS has been limited by existing gene synthesis techniques, restricting its analysis to single or a few amino acid mutations in only a small number of proteins (*17*) and being unable to probe the diversity of protein families that consist of thousands of members within a complex global landscape. This is due to several challenges faced by traditional gene synthesis techniques, including sequence complexity (*18*), length limitations imposed by phosphoramidite chemistry, which restricts oligonucleotide synthesis to fewer than 300 bases (*19*, *20*), and difficulties in assembling full-length genes from shorter fragments (*21*). Additional hurdles include increasing error rates with longer sequences (*22*), limited high-throughput capacity (*23*), and high costs associated with large-scale gene libraries (*24*).

To overcome these limitations, we developed a synthetic metagenomics approach to systematically reconstruct and functionally assay the DHFR protein family. While metagenomic sequencing has uncovered a vast reservoir of microbial genes (*25*), functional characterization remains limited by the difficulty of culturing native organisms and expressing heterologous sequences (*26*, *27*). To bypass these barriers, we synthesized a phylogenetically diverse library of DHFR homologs, most of which were mined from host-associated metagenomic datasets and annotated with the DHFR domain [InterPro: IPR012259 (*28*)]. By expressing these synthetic genes in a standardized *Escherichia coli* host and measuring growth across a gradient of TMP concentrations, we directly assessed the functional consequences of metagenome-derived sequence variation. This strategy represents one of the first large-scale efforts to assemble and functionally characterize a protein family from metagenomic data, enabling a systematic exploration of how natural sequence diversity in DHFR contributes to antibiotic resistance.

To implement this strategy at scale, we used DropSynth, a previously developed, multiplexed gene synthesis platform designed for constructing large, pooled libraries of full-length genes (*29*–*31*). DropSynth overcomes many limitations of traditional synthesis through optimized oligonucleotide design, droplet-based compartmentalization, and highly parallelized emulsion assembly. In this method, microarray-derived oligonucleotides are distributed into water-in-oil droplets, each containing the specific fragments needed to assemble a single gene. This physical separation prevents cross-hybridization between different gene constructs, enabling accurate assembly of thousands of sequences in parallel. Although some random mutations may be introduced during oligo synthesis or assembly, these can be harnessed to expand functional diversity and investigate local sequence constraints (*29*). The resulting library contains thousands of DHFR homologs and tens of thousands of unique mutant sequence variants, each tagged with a random barcode to facilitate pooled transformation, fitness screening, and recovery. The integration of DropSynth with a synthetic metagenomics framework provides a powerful and scalable solution for functionally profiling native sequence diversity in a controlled, high-throughput manner.

In this study, we combined DropSynth technology with broad mutational scanning (BMS), a high-throughput method for profiling the fitness of thousands of homologous protein variants in parallel (*29*), to map the functional landscape of diverse DHFR homologs at scale. Building on previous work where we designed and assembled two codon-optimized versions of a 1536-member DHFR homolog library (*30*), we pursued four primary objectives: (i) to demonstrate the effective use of DropSynth-assembled DHFR libraries in complementing metabolic function in an *E. coli* knockout strain, (ii) to evaluate the ability of DHFR homologs from diverse evolutionary lineages to confer resistance to the antibiotic TMP, (iii) to identify specific mutations in DHFR homologs from pathogenic bacterial species that contribute to antibiotic resistance, and (iv) to validate our multiplexed assay results using dial-out polymerase chain reaction (PCR) (*32*). To achieve these goals, we cloned the DHFR libraries into barcoded expression plasmids, transformed them into *E. coli ER2566* ∆*folA∆thyA* cells (*33*), and screened for their ability to complement the *folA* knockout phenotype while testing resistance to TMP across a concentration gradient. Dial-out PCR enabled independent testing of select homologs, allowing us to assess variant performance without competitive interference from other homologs to validate our pooled fitness results. This proof-of-concept study demonstrates the power of DropSynth-assembled libraries in multiplexed functional assays, showcasing our synthetic metagenomics approach for constructing and functionally characterizing a diverse pool of DNA sequences. This strategy enables rational exploration of sequence-function relationships within the DHFR protein family at an unprecedented scale. It also provides a rich dataset for training machine learning (ML) models in generative structure-function prediction. Ultimately, this work advances our understanding of the evolutionary mechanisms underlying antibiotic resistance in DHFR, informing strategies to address this critical health challenge.

## RESULTS

### DropSynth assembly of DHFR gene homologs

**Fig. 1.**
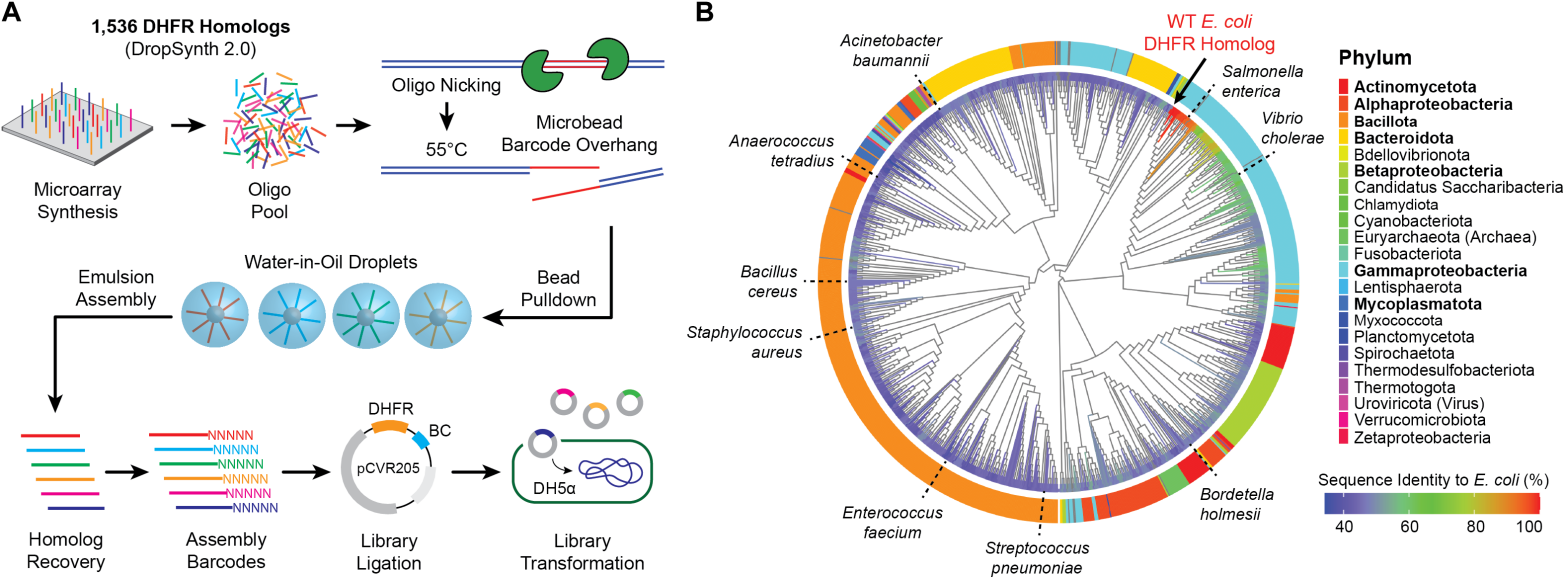
DropSynth assembly of 1536-DHFR gene homologs. (**A**) Schematic of the DropSynth 2.0 process: Microarray-derived oligos are amplified and pulled down by barcoded beads that selectively capture oligos for gene assembly. Beads are emulsified in droplets with genes assembled via polymerase cycling assembly. After breaking the emulsion, genes are barcoded, ligated into an expression plasmid, and transformed into DH5α cells. (**B**) Maximum likelihood phylogenetic tree of 1208 successfully assembled DHFR homologs from the 1536-member library. Branch color indicates percent sequence identity to wild-type (WT) *E. coli* DHFR, with taxonomic diversity annotated at the class level for the phylum Pseudomonadota. DHFR homologs from known pathogenic bacterial species are annotated for reference. Phyla in bold represent the highest unique sequence abundances in the DHFR library.

We previously designed and assembled two codon versions of a 1536-member library of DHFR homologs (*30*). The codon 1 library was designed using weighted random assignment based on *E. coli* codon usage to favor expression, while the codon 2 library maximized synonymous sequence divergence using a lookup table. Rare codons were excluded from both designs. While codon 1 may generally support higher expression in the *E. coli folA* knockout host, neither scheme is inherently optimal across all homologs, and expression differences are likely to be protein specific. These libraries were assembled using our top-performing polymerase, KAPA HiFi, barcoded with a random 20-nucleotide assembly barcode, ligated into an expression plasmid (pCVR205), and transformed into DH5α cells to maximize ligation efficiency and library yield (Fig. 1A; see Materials and Methods for more details). Using our optimized protocol, we assembled 1208 unique DHFR homologs across both codon versions, spanning a broad phylogenetic range with varying evolutionary distances from *E. coli* (Fig. 1B). Most homologs were derived from bacterial species, spanning 17 phyla, 30 classes, 71 orders, 153 families, 320 genera, and 759 species. These include representatives from major groups such as Actinomycetota, Bacillota, Bacteroidota, Mycoplasmatota, and classes of Pseudomonadota (table S1). Our library design intentionally incorporates DHFR homologs from clinically important pathogens, including members of the ESKAPE group (Fig. 1B) (*34*, *35*). We also included a small subset of archaeal and viral DHFR homologs as outgroup comparisons to probe the evolutionary limits of functional compatibility in the *E. coli* model.

### Complementation assay of DHFR homologs and mutants

Our first objective was to demonstrate how DropSynth-assembled libraries can be seamlessly integrated into multiplex functional assays. To this end, we evaluated our phylogenetically diverse library of DHFR homologs for their ability to functionally complement DHFR activity in *E. coli ER2566* ∆*folA*∆*thyA* cells. This knockout strain—derived from the *E. coli* B lineage commonly used in protein expression studies—lacks both the chromosomal *folA* gene (encoding DHFR) and *thyA* (encoding thymidylate synthase), rendering it auxotrophic for thymidine and completely dependent on exogenous DHFR activity for survival in minimal medium (*36*). Each homolog was expressed from the pCVR205 plasmid, derived from the SMT205 backbone, which was engineered with a T7 promoter and weak ribosome binding site to drive DHFR expression at ~10% of native protein levels (*33*). This subsaturating expression context was intentionally designed to enhance assay sensitivity to DHFR catalytic efficiency, enabling the resolution of both partial loss-of-function and GOF variants that would otherwise be masked under higher expression conditions (*33*). In this system, only DHFR variants with sufficient catalytic activity to sustain folate metabolism can support cell growth. In addition, the plasmid expresses *thyA* under the same promoter to complement the knockout strain’s thymidylate synthase deficiency, minimizing the risk of folate pathway imbalance and toxicity associated with DHFR overexpression (*37*).

**Fig. 2.**
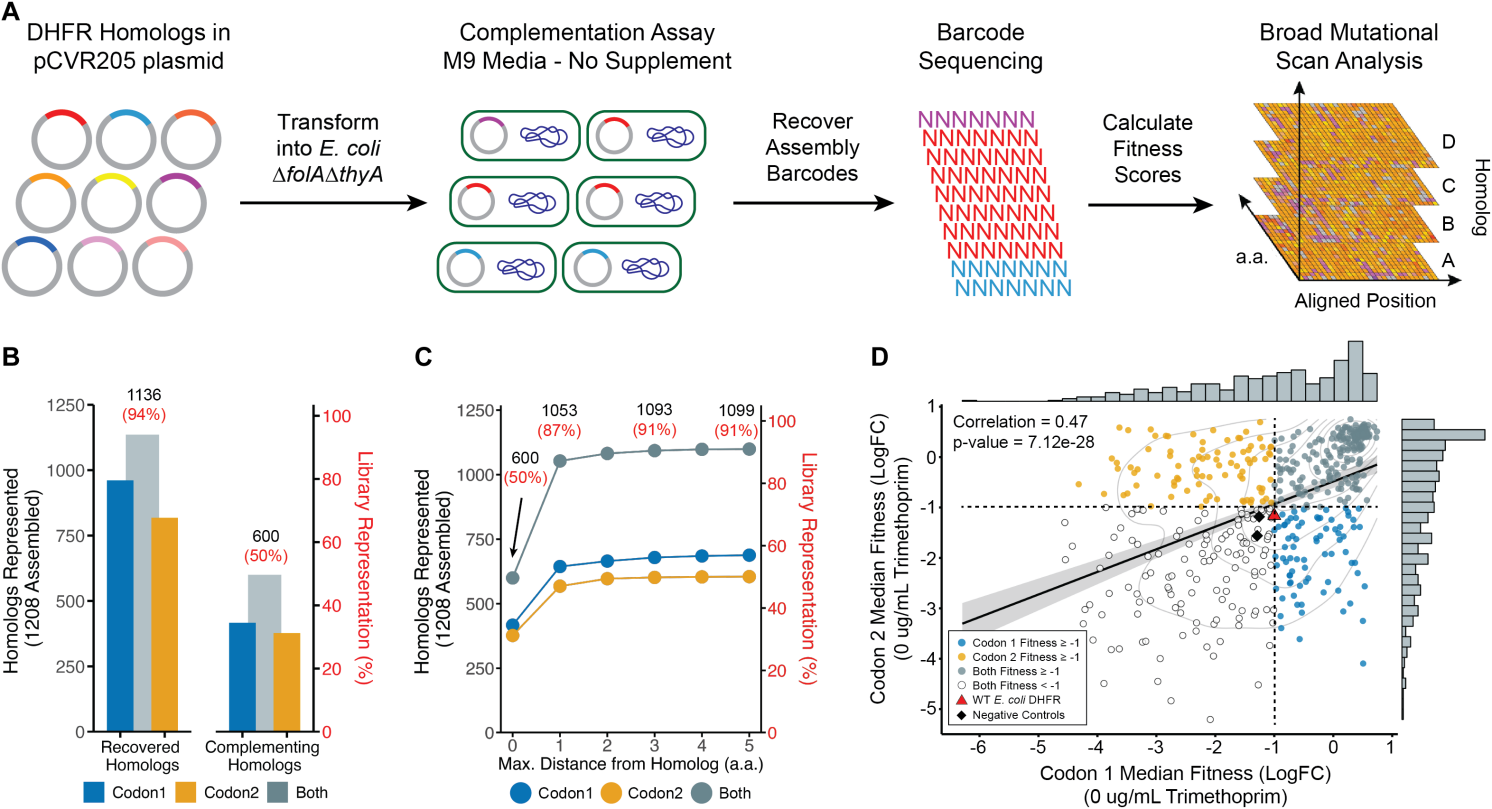
Complementation assay of DHFR homologs and mutants. (**A**) Barcoded DHFR homologs in the pCVR205 plasmid were transformed into folate-deficient *E. coli ER2566* ∆*folA∆thyA* cells using a pooled complementation assay with growth on solid medium. Fitness scores were based on the log_2_ fold change in barcode recovery between supplemented and nonsupplemented conditions. Sequence alignment enabled mapping fitness onto the WT *E. coli* DHFR sequence. aa, amino acid. (**B**) Of the 1208 DropSynth-assembled homologs, we recovered 961 from codon 1 and 818 from codon 2 with a minimum of 10 sequence reads per barcode, with 1136 homologs represented across both codon libraries under complementation conditions (94% total library coverage). Selecting homologs showing some degree of complementation (fitness ≥ −1) revealed 416 homologs from codon 1 (34%) and 377 homologs from codon 2 (31%), totaling 600 unique homologs capable of complementation across both codon versions, achieving 50% coverage of the total library. (**C**) Adding mutant assemblies (within five–amino acid distance), we recovered 1099 homologs capable of complementation (fitness ≥ −1), achieving 91% of total library coverage. (**D**) Fitness values for 485 homologs shared between codon versions showed a significant correlation (ρ = 0.47, Pearson), with points representing fitness in codon 1 (blue), codon 2 (orange), or both (teal). Uncolored points represent homologs with low fitness during complementation (fitness < −1). WT *E. coli* DHFR and negative controls (D27N and mCherry) are plotted for reference. Dashed lines extending from the *x* and *y* axes at −1 indicate the minimum fitness threshold for complementation in both codon libraries. FC, fold change.

Following transformation, cells were initially recovered on LB agar supplemented with chloramphenicol and thymidine. After pregrowth in M9 medium supplemented with thymidine and folate, cells were washed and transferred to nonsupplemented M9 minimal medium (i.e., no thymidine or folate) and incubated at 37°C for 22 hours (Fig. 2A). Under these stringent conditions, survival depends on the ability of the introduced DHFR homolog to support folate metabolism. To account for sequencing depth variation, we normalized barcode counts to the total number of reads under the M9-supplemented condition. Fitness was calculated as the log_2_ fold change in normalized barcode abundance between supplemented and nonsupplemented conditions. This complementation assay establishes a functional baseline for each homolog and enables robust comparison of relative growth across the TMP selection gradient (see Materials and Methods for details).

Barcodes were previously mapped to their corresponding DHFR variants using Illumina MiSeq sequencing (*30*). Illumina NovaSeq sequencing of the assembly barcodes recovered from all nine experimental growth conditions (LB medium, supplemented M9 medium, nonsupplemented M9 medium, and six TMP concentrations in nonsupplemented M9 medium) generated 1,010,919,383 sequence reads across both codon version libraries. These reads corresponded to 3,947,715 assembly barcodes with at least one read count (table S2).

To ensure downstream data quality, we applied a two-tier barcode filtering strategy. First, we retained only barcodes with ≥10 reads under at least one experimental condition to minimize sequencing noise. Second, we included only homologs with ≥5 high-confidence barcodes to ensure robust replication. This filtering yielded 227,451 unique mapped barcodes under the nonsupplemented complementation condition, representing 56.4% of the 403,154 total unique barcodes across both codon libraries. Of these, 65% (147,868) corresponded to perfect assemblies, while 35% (79,617) represented mutant variants (fig. S1). Collectively, the mapped barcodes spanned 1136 unique homologs, covering 94% of the 1208 successfully assembled designs from the original library (Fig. 2B). A detailed flowchart of the data filtering and retention steps is provided in fig. S2. In practice, our dataset exceeded these filtering thresholds by a wide margin. The median number of raw barcodes per homolog was 227 for codon 1 and 213 for codon 2. After filtering, the median number of high-confidence barcodes retained per homolog was 39 in codon 1 and 46 in codon 2. On average, 76 barcodes were retained per homolog in codon 1 and 131 in codon 2, with many homologs represented by more than 100 high-confidence barcodes (table S3).

After applying our two-tier filtering strategy to retain only perfect assembly homologs with at least five unique high-confidence barcodes, we identified 797 homologs in the codon 1 library and 666 in the codon 2 library under complementation conditions, representing 970 unique homologs across both codon versions (fig. S2 and table S4). We also retained 59,763 mutant variants associated with codon 1 and 49,691 with codon 2, for a total of 109,454 unique mutants. Of these, 12,274 (codon 1) and 16,060 (codon 2) contained ≤5 amino acid substitutions relative to the intended sequence, yielding a final set of 28,334 high-confidence mutant variants used for downstream analysis. Each designed homolog had a median of 18 associated mutant variants across both codon versions. Fitness scores were calculated for both designed and mutant variants as the log_2_ fold change in barcode recovery between M9-supplemented and nonsupplemented conditions (Fig. 2A).

Among perfect assembly homologs passing quality filters, 416 from codon 1 and 377 from codon 2 showed complementation (fitness ≥ −1), representing 600 unique homologs and ~50% of the DropSynth-assembled library (Fig. 2B). Including high-confidence mutant variants (≤5 amino acid substitutions from the designed homolog), we recovered 1099 unique homologs capable of complementation across both codon versions, covering 91% of library members passing quality filters (Fig. 2C). Most of these variants (1053) differed by only a single amino acid, highlighting the functional tolerance of many homologs to limited mutation. In contrast, highly mutated variants with more than 50 substitutions, often resulting from frameshift-inducing deletions, exhibited low false-positive rates (0.6% in codon 1 and 1.3% in codon 2; table S5). Mutant assemblies with more than five amino acid changes were excluded to avoid artifacts from excessive mutational burden and ensure fitness reflected the intended sequences. The −1 fitness threshold, reflecting a twofold drop from the M9-supplemented condition, serves as a conservative complementation cutoff, supported by negative controls that fall just below this value (Fig. 2D).

Of the 970 high-quality, perfect assembly DHFR homologs retained under complementation conditions, 493 were shared between the codon 1 and codon 2 libraries (table S4), with significantly correlated fitness values (Pearson ρ = 0.47, *P* = 7.12 × 10^−28^; Fig. 2D). Among the 493 shared homologs, 202 (41%) complemented in both libraries (fitness ≥ −1), while 77 (16%) and 86 (17%) complemented only in codon 1 or codon 2, respectively; 128 (26%) failed to complement in either. The wild-type (WT) *E. coli* DHFR positive control showed lower than expected fitness across codon versions, likely due to the ~10% expression level of our assay in nonsupplemented M9 medium. Negative controls, including the catalytically impaired D27N variant (*38*) and non-DHFR mCherry protein (*39*), exhibited low fitness in both libraries (Fig. 2D). These null controls provide critical benchmarks for interpreting homolog fitness and help define the lower bound of complementation. As expected, most archaeal and viral homologs did not complement (table S6). However, one viral (*Aeromonas* phage Aeh1) and two archaeal (Euryarchaeota) variants supported growth in *E. coli* (fitness ≥ −1), demonstrating rare but possible cross-domain compatibility.

**Fig. 3.**
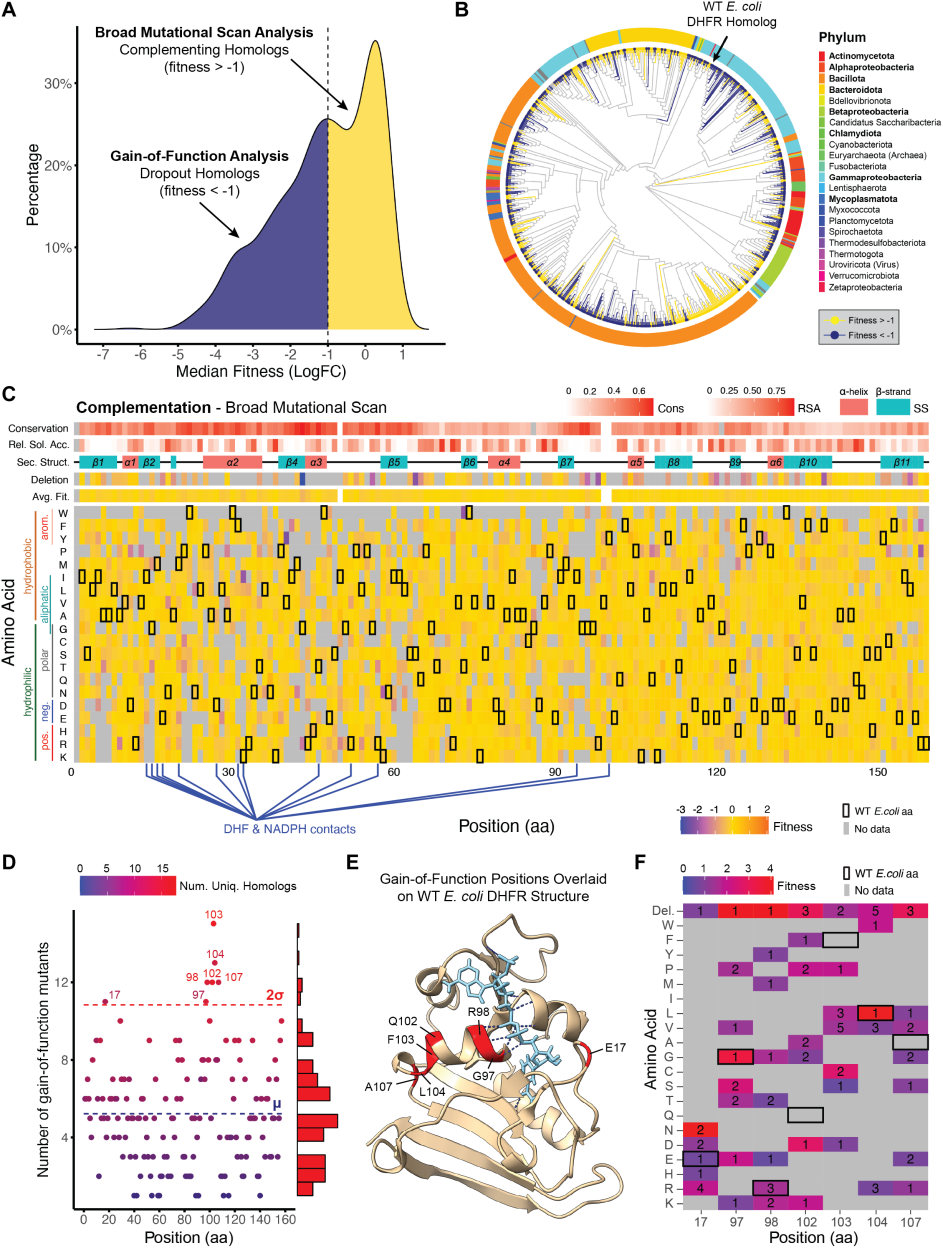
BMS and GOF mutations in the complementation assay. (**A**) Fitness distribution of 797 DHFR homologs from the codon 1 complementation assay, with 416 complementing homologs (fitness ≥ −1; yellow) used in BMS and 381 dropout homologs (fitness < −1; blue) used in GOF analysis. (**B**) Phylogenetic tree of 797 DHFR homologs, highlighting complementing and dropout homologs. (**C**) Fitness landscape of 416 complementing homologs and 5419 associated mutants (≤5 substitutions), mapped onto the aligned *E. coli* DHFR sequence. Heatmap *x* axis shows residue positions; *y* axis indicates median fitness of each amino acid recovered (log_2_ fold change). Annotation tracks above the plot show secondary structure (magenta = helices and cyan = sheets), relative solvent accessibility (darker = more buried), and sequence conservation (darker = more conserved). WT residues are marked in black. Gray squares indicate no amino acid data. Sec. struct., secondary structure (SS); Rel. sol. acc., relative solvent accessibility (RSA); Cons, conservation; arom., aromatic; Avg. fit., average fitness; Pos., positive; Neg., negative. (**D**) GOF analysis identified 476 mutants (fitness ≥ −1) associated with 196 dropout homologs, projected onto the WT *E. coli* DHFR sequence, highlighting seven statistically significant positions (residues 17, 97, 98, 102, 103, 104, and 107) where mutation frequency exceeded the mean plus two SDs (μ + 2σ) across all aligned positions. Num. uniq., number of unique. (**E**) WT *E. coli* DHFR structure (4KJK) with GOF residues shaded in red, reduced form of nicotinamide adenine dinucleotide phosphate (NADPH) cofactor in cyan, and H^+^ bonds in blue. (**F**) Heatmap showing mean fitness (color shading) and the number of observed GOF mutants (numbers) at each identified GOF position. Del., deletion.

The fitness distribution of perfect assembly homologs under complementation conditions separates into two groups: those capable of complementation (fitness ≥ −1), included in the BMS analysis, and dropout homologs (fitness < −1), analyzed for GOF mutations (Fig. 3A). Focusing on codon 1 homologs optimized for *E. coli* expression, we found that 416 of 797 (52%) complemented, while 381 (48%) did not. These homologs span the phylogenetic tree with only modest clade-level clustering (Fig. 3B). Notably, some close *E. coli* relatives failed to complement, likely due to the assay’s subsaturating expression (~10% of native levels), which increases sensitivity to variation in expression, stability, or catalytic activity (*33*, *37*, *40*). Conversely, many phylogenetically distant homologs complemented successfully despite only ~46% median sequence identity with *E. coli* DHFR (Fig. 1B), underscoring the deep evolutionary conservation of DHFR function (Fig. 3B and fig. S3).

For the BMS analysis, we selected 416 complementing homologs from the codon 1 library (fitness ≥ −1) along with 5419 mapped mutants carrying up to five amino acid substitutions. Following multiple sequence alignment, we calculated the median fitness for each amino acid at each aligned position. These data were collapsed and projected onto the *E. coli* DHFR sequence (Fig. 3C), generating a baseline positional fitness landscape similar to conventional DMS (*41*, *42*). Because the analysis includes only functional homologs, positional fitness values are relatively uniform, reflecting constrained variation across compatible sequences. This serves as a baseline for interpreting shifts in positional fitness under increasing TMP concentrations, where site-specific selection pressures will likely become more apparent. Average BMS fitness was negatively correlated with positional conservation (ρ = −0.51, Pearson; *P* = 6.54 × 10^−9^), indicating that highly conserved sites are more mutationally constrained (fig. S4A). Buried residues showed only a modest reduction in average fitness compared to solvent-accessible residues (ρ = 0.24, Pearson; *P* = 0.003), based on the Dictionary of Secondary Structure in Proteins (DSSP) analysis of the 1H1T crystal structure (fig. S4B). In addition, positional fitness was positively correlated with mutational coverage (ρ = 0.49, Pearson; *P* = 8.63 × 10^−11^), as low-fitness variants were more likely to be filtered out, slightly inflating average scores (fig. S4C). Notably, no significant differences in average fitness were observed across secondary structure classes such as helices, loops, and strands (fig. S4D).

In the GOF analysis, we identified 476 single–amino-acid mutants (fitness ≥ −1) that restored function to 196 of the 381 noncomplementing (dropout) homologs. These dropouts showed fitness levels comparable to the negative controls, D27N and mCherry, which lack complementation activity and define the lower bound for functional rescue in *E. coli* (Fig. 2D). Notably, we observed some selective pressure under the M9-supplemented condition (which contains thymidine). To prioritize GOF, we calculated fitness as the log_2_ ratio between the nonsupplemented and supplemented conditions. This approach increased sensitivity to subtle complementation by narrowing the dynamic range between functional and nonfunctional variants (fig. S5), making negative controls appear more fit (D27N = −1.3 and mCherry = −1.3) than under LB-baseline calculations (D27N = −4.2 and mCherry = −3.3). Using this framework, we identified seven positions in the *E. coli* DHFR sequence (residues 17, 97, 98, 102, 103, 104, and 107) where GOF mutations were enriched, occurring more frequently than expected based on a threshold of the mean plus two SDs across all aligned positions (μ + 2σ; Fig. 3D). These sites likely contribute to functional rescue by enhancing catalytic activity, stability, or expression in the *E. coli* system. When mapped onto the *E. coli* DHFR structure [4KJK; Protein Data Bank (PDB)] (*43*), six of the seven residues clustered within a short α helix adjacent to the reduced form of nicotinamide adenine dinucleotide phosphate (NADPH) binding pocket (Fig. 3E), including residues 97 and 98, which coordinate the nicotinamide ring, and residue 17 within the Met20 loop, a catalytic element essential for folate reduction (*44*). Multiple distinct substitutions, including numerous deletions, restored fitness at these positions, highlighting both their sensitivity to mutation and potential for adaptive, GOF rescue (Fig. 3F).

### TMP resistance of DHFR homologs and mutants

**Fig. 4.**
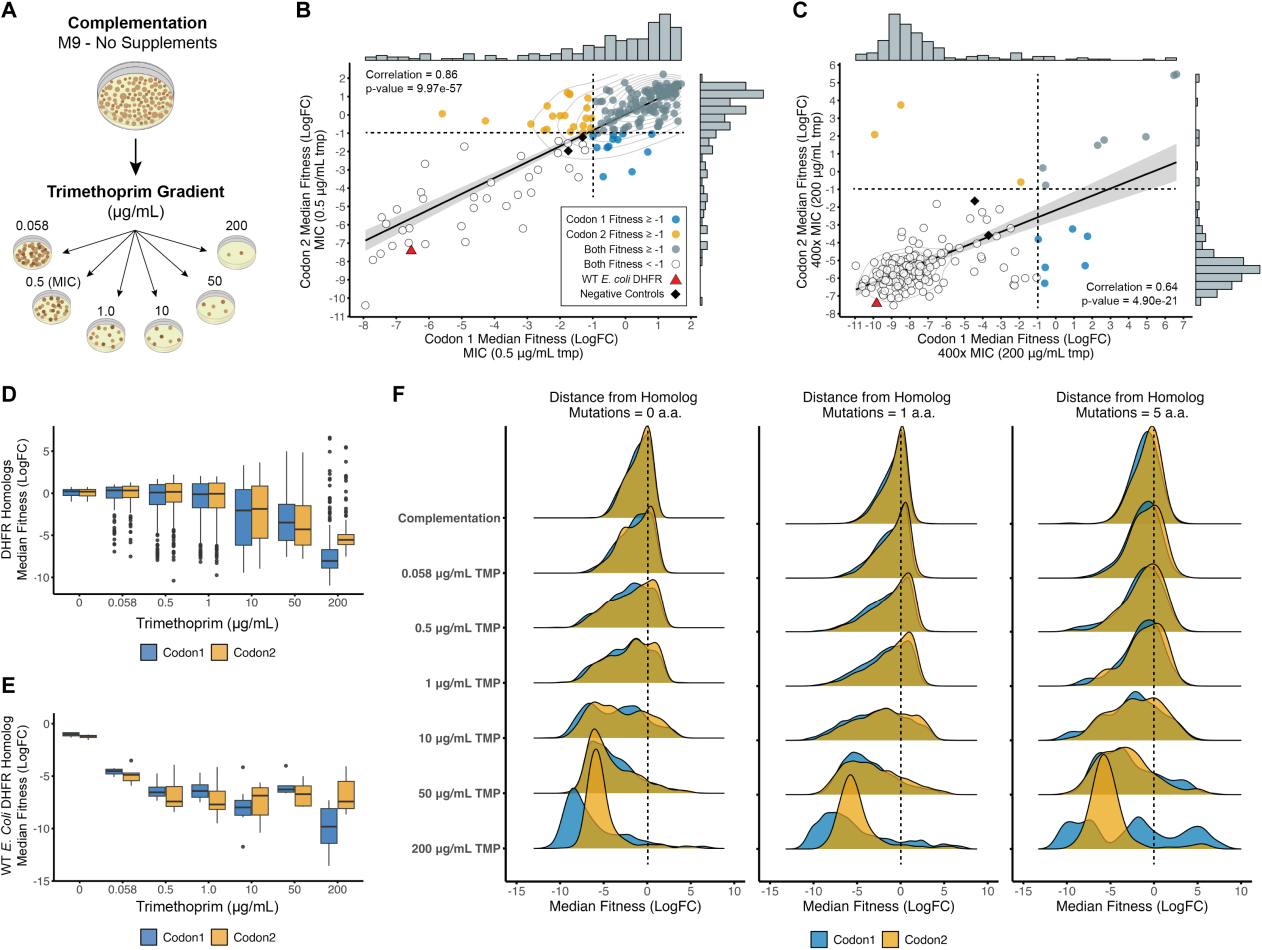
TMP fitness assay of DHFR homologs and mutants. (**A**) Overview showing how *E. coli ER2566 ∆folA∆thyA* cells containing unique barcoded DHFR homologs were grown across a TMP gradient. Fitness scores were calculated on the basis of the log_2_ fold change in barcode sequence recovery between TMP-treated and supplemented conditions. (**B**) At MIC (0.5 μg/ml), fitness values for 190 shared homologs showed a strong positive correlation (ρ = 0.86, Pearson) between codon versions, with many homologs demonstrating resistance (fitness ≥ −1). (**C**) At 400× MIC (200 μg/ml), fitness values for 174 shared homologs showed a significant positive correlation (ρ = 0.64, Pearson) between codon versions, although most homologs could not maintain function (fitness < −1). Points are colored on the basis of fitness for codon-specific homologs, shared homologs, or those with low fitness. The WT *E. coli* DHFR homolog and negative controls are plotted for reference. Dashed lines denote the minimum fitness thresholds for TMP resistance. (**D**) Box plot showing homolog fitness declined consistently with increasing TMP concentration across the gradient for both codon versions. (**E**) Box plot showing WT *E. coli* DHFR homolog (positive control) fitness declined similarly across both codon versions. (**F**) Ridge plots show that the TMP gradient affected the median fitness distributions for perfect assemblies and mutants with one or five amino acid differences, with survival decreasing as TMP concentration increased.

Following complementation, our next objective was to test whether DropSynth-assembled libraries could maintain metabolic function under TMP exposure. To assess antibiotic resistance, we exposed *E. coli ER2566* ∆*folA∆thyA* cells containing barcoded DHFR plasmids to a TMP gradient ranging from 0 to 400× the minimal inhibitory concentration (MIC) of 0.5 μg/ml in minimal M9 medium. TMP concentrations included 0 (complementation), 0.058, 0.5 (MIC), 1.0, 10, 50, and 200 (400× MIC) μg/ml (Fig. 4A). Plasmid barcodes from cells surviving each TMP condition were recovered and sequenced to quantify the abundance of each unique DHFR variant across the TMP gradient. This approach enabled us to identify resistant (i.e., high-fitness) variants that thrived under increasing antibiotic pressure and susceptible (i.e., low-fitness) variants that were progressively depleted with increasing TMP concentrations.

Among the 600 perfect assembly homologs that complemented across both codon versions (fitness ≥ −1; see Fig. 2B for reference), only 190 shared homologs were recovered at the MIC level. These shared homologs showed a significant positive fitness correlation (ρ = 0.86, Pearson; *P* = 9.97 × 10^−57^), with most maintaining metabolic function (fitness ≥ −1) at MIC selection (Fig. 4B). At 400× MIC (200 μg/ml), we recovered 174 shared homologs that displayed a positive but lower fitness correlation than at MIC (ρ = 0.64, Pearson; *P* = 4.90 × 10^−21^), with most unable to maintain function (fitness < −1) (Fig. 4C). Notably, we identified seven shared homologs and nine additional codon-specific homologs capable of maintaining function (fitness ≥ −1) under high TMP, indicating resistance to TMP (Fig. 4C). These include members of Bacillota (e.g., *Clostridium* spp.), Bacteroidota (*Galbibacter marinus* and *Niabella soli*), and Pseudomonadota (e.g., *Neisseria flavescens*) (table S7). We also recovered homologs with higher median fitness [maximum (max.) fitness = 6.6] than observed under the complementation condition (max. fitness = 0.7; see Fig. 2D). This is likely due to the compositional nature of the data: As many variants die off, the relative fraction of tolerant variants increases, resulting in higher enrichment scores. Pearson fitness correlations for homologs shared between codon versions under each TMP condition are provided in the Supplementary Materials (see fig. S6).

Across the TMP gradient, increasing antibiotic concentrations consistently decreased median homolog fitness for both codon version libraries (Fig. 4D). This trend demonstrates that even high-fitness homologs experience reduced viability with increasing TMP inhibition. The WT *E. coli* DHFR, included in each library as a positive control, showed a consistent fitness decline across the TMP gradient, reflecting the substantial metabolic stress imposed by TMP (Fig. 4E). In contrast, the fitness profiles of the negative controls (D27N and mCherry), which are unaffected by TMP, are shown alongside the WT for comparison (fig. S7). Similarly, the few archaeal and viral outgroup homologs capable of complementation also exhibited decreasing fitness with increasing TMP concentrations (fig. S8). When examining DHFR variants with either a single–amino-acid change or up to five amino acid changes, we observed declining fitness values with increasing TMP concentration that were comparable to those of the perfect assembly homologs (i.e., zero mutations), consistent across both codon versions (Fig. 4F). However, the ridge plots reveal a nuanced, codon-specific response at the highest TMP concentration (200 μg/ml). Homologs with up to five amino acid differences in the codon 1 library showed substantially divergent resistance outcomes: Where some displayed extremely low fitness (<−5), others experienced moderate declines (fitness between −1 and −5), and a subset achieved strong positive fitness (>5). This variation suggests that specific protein mutations in the codon 1 library may provide certain homologs with enhanced resistance to high TMP inhibition, indicating potential adaptive advantages unique to this codon version (see fig. S9 for more details).

**Fig. 5.**
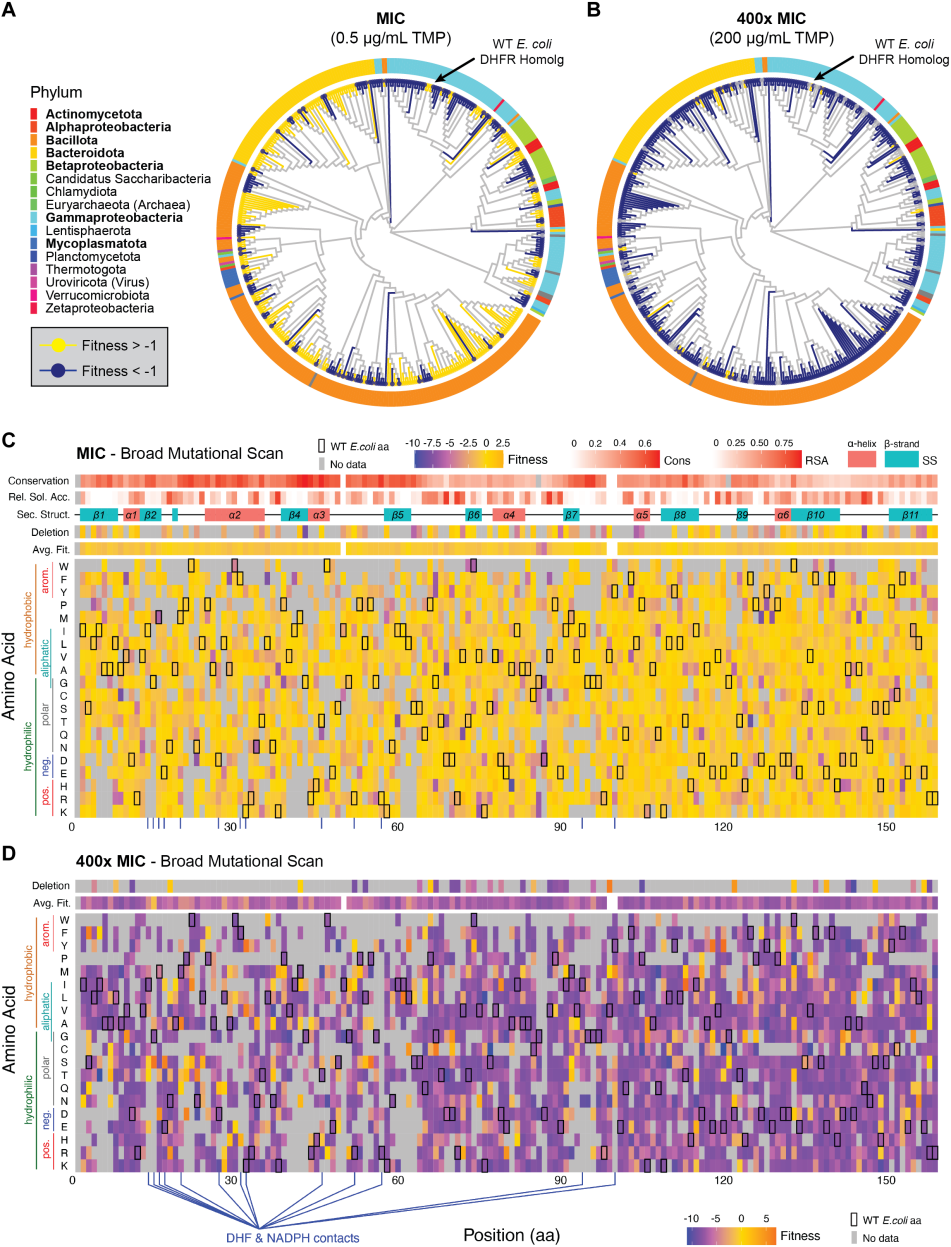
TMP fitness: Phylogenetic distribution and BMS analysis. (**A**) Maximum likelihood phylogenetic tree of 416 DHFR homologs, showing TMP-resistant homologs (fitness > −1) at MIC [TMP (0.5 μg/ml)] in yellow and nonresistant homologs (fitness < −1) in blue. Some degree of clustering can be observed. (**B**) Maximum likelihood phylogenetic tree of 416 DHFR homologs at 400× MIC [TMP (200 μg/ml)], with resistant homologs in yellow and nonresistant in blue. (**C**) Fitness landscape of 416 homologs and their associated mutants (within a five–amino-acid range) mapped onto the WT *E. coli* DHFR sequence at MIC, with each homolog initially capable of complementing function in nonsupplemented medium. (**D**) Fitness landscape of 416 homologs and their associated mutants (within a five–amino-acid distance) mapped onto the WT *E. coli* DHFR sequence at 400× MIC. Both heatmaps show the average fitness of an amino acid substitution at each position along the protein sequence, with WT residues marked by black squares, and data on average fitness, deletions, secondary structure, relative solvent accessibility, and conservation included. Gray squares indicate no amino acid data.

To analyze the mutational fitness landscape across the TMP gradient, we again focused on homologs from the codon 1 library optimized for *E. coli* expression. Of the 416 homologs capable of complementation under nonsupplemented conditions (fitness ≥ −1; see Fig. 3A for reference), we observed a decline in the number of homologs resistant to TMP inhibition, as the antibiotic concentration increased across the gradient (see fig. S10). Specifically, 318 homologs (76%) were resistant (fitness ≥ −1) at TMP (0.058 μg/ml), 246 homologs (59%) at MIC (0.5 μg/ml), 226 homologs (54%) at 1.0 μg/ml, 128 homologs (31%) at 10 μg/ml, 80 homologs (19%) at 50 μg/ml, and only 27 homologs (7%) remained resistant at TMP (200 μg/ml) (table S8). Although most homologs were resistant to TMP at the MIC level (59%), the absolute differences in fitness values between homolog pairs were not evenly distributed across the phylogenetic tree, exhibiting significant variation based on evolutionary distance (Fig. 5A). A Wilcoxon rank sum test indicated a highly significant difference in fitness variation (*W* = 3.2 × 10^8^ and *P* < 2.2 × 10^−16^), supported by Welch’s *t* test, which showed a lower mean absolute fitness difference for closely related homologs compared to distantly related homologs (*t* = −63.5, df = 7,020, and *P* < 2.2 × 10^−16^; table S9). These results suggest that evolutionary relatedness significantly influences homolog fitness similarity under MIC selection, with closely related species tending to exhibit more similar fitness responses to TMP treatment.

At the high TMP concentration (200 μg/ml), only 27 homologs in the codon 1 library were able to maintain function while resisting TMP inhibition (fitness ≥ −1; table S8). As with the MIC results, fitness differences between homolog pairs at this higher concentration varied significantly across the phylogenetic tree, influenced by evolutionary distance (Fig. 5B). Statistical support at the high TMP concentration was comparable to the MIC level, where the Wilcoxon rank sum test still indicated a highly significant difference in fitness variation (*W* = 2.8 × 10^8^ and *P* < 2.2 × 10^−16^), and Welch’s *t* test confirmed a lower mean absolute fitness difference for closely related homologs compared to distantly related homologs (*t* = −26.2, df = 6206, and *P* < 2.2 × 10^−16^; table S9). These findings reinforce the trend observed at the MIC, suggesting that evolutionary relatedness continues to influence fitness similarity, with closely related species showing more comparable fitness responses even at high TMP concentrations.

For the BMS analysis across the TMP gradient, we selected the same 416 homologs and their mutants (within a five–amino-acid distance) that successfully complemented function in nonsupplemented medium (fitness ≥ −1; see Fig. 3C for reference). This selection criterion focuses our analysis on functionally relevant variants under antibiotic pressure and enables a direct comparison of median fitness changes for each amino acid at each position across the gradient. These fitness scores are then mapped onto the *E. coli* DHFR sequence as a reference, contextualizing the results within a well-characterized model organism (Fig. 5, C and D). The resulting heatmaps illustrate the fitness landscape within the inherited sequence space, reflecting the natural sequence diversity of the DHFR protein family, as it has evolved across species. Each point on the heatmap represents the average effect of a specific amino acid–position combination, aggregated across diverse sequence backgrounds, offering insights into how sequence variation influences fitness.

Under MIC selection, most amino acid substitutions at each position across the DHFR protein sequence support sufficient fitness (≥−1) to maintain function (median fitness = −0.4; Fig. 5C). However, certain positions exhibit a median fitness well below the −1 threshold, indicating greater depletion and suggesting that mutations at these residues are less tolerated, leading to reduced survival rates. Notably, residue 85 (median fitness = −3.2; *t* test = 7.0, df = 52, and *P* = 5.43 × 10^−9^) and residue 86 (median fitness = −6.1; *t* = 6.1, df = 15, and *P* = 2.14 × 10^−5^) show significantly lower fitness compared to other positions (see fig. S11). An examination of the multiple sequence alignment used to map the high-dimensional data onto the *E. coli* reference revealed that positions 85 and 86 fall within gapped regions for most homologs, except for *E. coli*. This underscores a limitation of alignment-based dimensionality reduction, where gaps in homologous sequences can obscure functional constraints and introduce artifacts into fitness estimates. At 400× MIC selection, most amino acid substitutions across the protein sequence did not support sufficient fitness (≥−1) to maintain DHFR function, with a median fitness of −8.2 (Fig. 5D).

As TMP concentrations increased across the experimental gradient, amino acid diversity at each DHFR position declined, reflecting the progressive elimination of low-fitness variants under increasing antibiotic pressure. Specifically, the average number of tolerated amino acids per position decreased from 17 at complementation to 16 at MIC and only 14 at 400× MIC. This trend highlights the selective pressure exerted by TMP, which progressively narrows the range of functional substitutions that can maintain DHFR activity. For example, at residue 85, the number of tolerated amino acids decreased from 10 under complementation to 7 at 400× MIC. Similarly, residue 86 showed a sharper decline in tolerated substitutions, dropping from seven to three, indicating that previously functional substitutions become increasingly incompatible with DHFR activity under higher TMP concentrations. This reduced tolerance highlights critical residues essential for the enzyme’s ability to withstand antibiotic stress, as evidenced by the loss of less-fit variants.

### Resistance-conferring mutations in DHFR homologs from pathogenic bacteria

Our third objective was to identify specific mutations within DHFR homologs derived from pathogenic bacterial species that contribute to antibiotic resistance. To achieve this, we designed our DHFR library to include a broad range of obligate and opportunistic pathogens (Fig. 1B), including most members of the “ESKAPE” group (*34*). These bacteria, including *Enterococcus faecium*, *Klebsiella pneumoniae*, *Acinetobacter baumannii*, and *Pseudomonas aeruginosa*, are major contributors to antibiotic-resistant infections in clinical settings (*35*). Our library also includes DHFR sequences from clinically relevant pathogens with varying levels of susceptibility to TMP, including *Anaerococcus tetradius*, *Bacillus cereus*, *Haemophilus influenzae, Salmonella enterica*, *Staphylococcus aureus*, *Streptococcus pneumoniae*, and *Vibrio cholerae* (*3*). This diverse collection, sourced from host-associated metagenomes, enabled the first unified assessment of resistance-conferring mutations across DHFR homologs from pathogenic sources.

While our DHFR library includes at least one homolog from each of the pathogens listed above, some variants failed to complement function in the *E. coli* ∆*folA∆thyA* cells, resulting in a lack of fitness data for those homologs. Nevertheless, we successfully recovered fitness data for eight well-characterized DHFR homologs derived from pathogenic bacterial species, enabling us to demonstrate the effectiveness of our synthetic metagenomics approach in identifying resistance-conferring mutations (see fig. S12). Among these, we focused on two homologs, *B. cereus* and *S. pneumoniae*, which exhibited numerous mutant variants with diverse fitness responses across the TMP gradient. This enabled us to identify specific amino acid substitutions associated with TMP resistance and gain insight into the molecular mechanisms driving resistance.

**Fig. 6.**
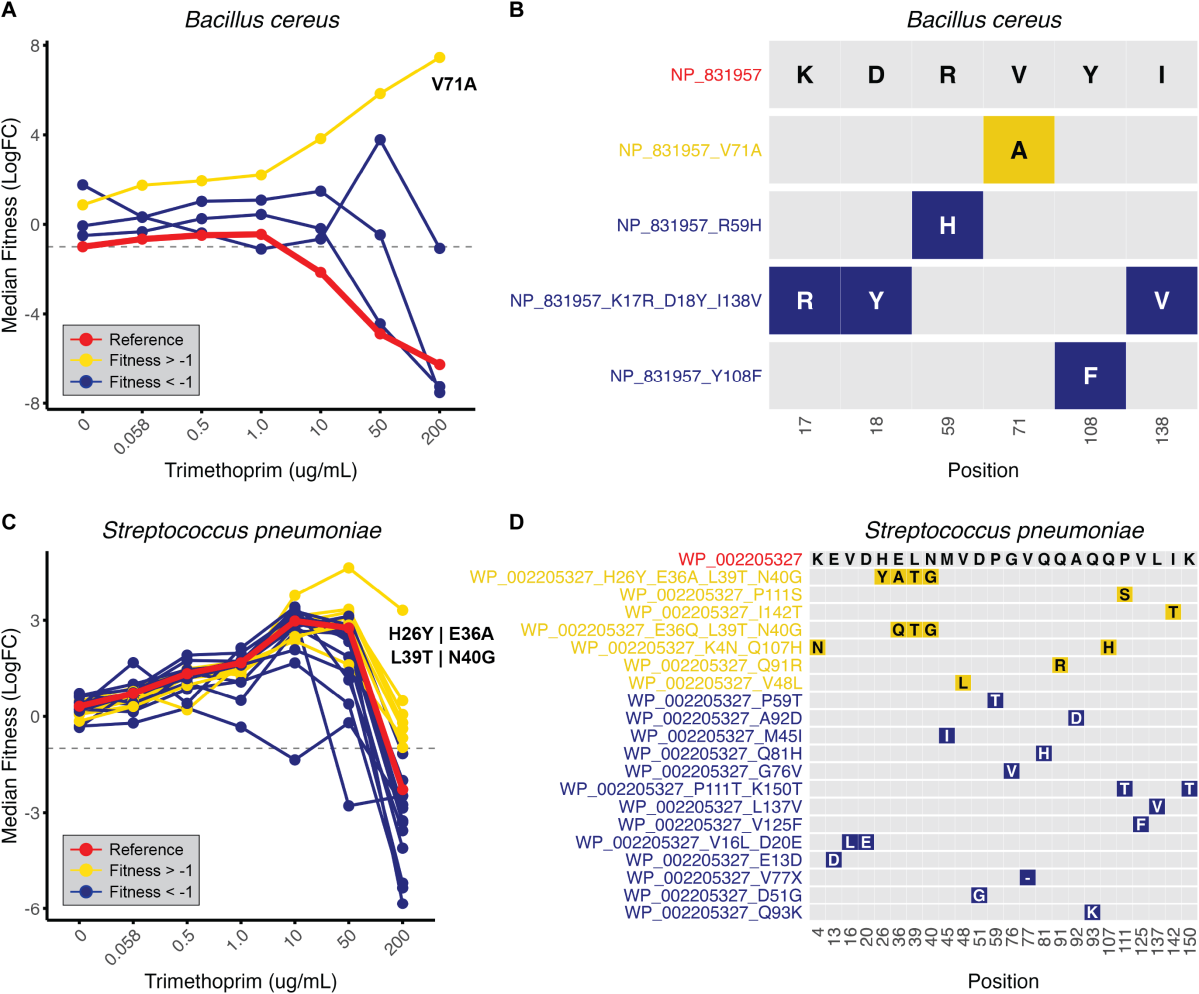
Resistance-conferring mutations in DHFR homologs from pathogenic bacteria. (**A**) Median fitness of *B. cereus* DHFR homolog across the TMP gradient, with the reference sequence shown in red, mutants classified as TMP-resistant [fitness ≥ −1 at TMP (200 μg/ml)] shown in yellow, and nonresistant mutants [fitness < −1 at TMP (200 μg/ml)] shown in blue. (**B**) Grid plot of amino acid substitutions in *B. cereus* (NP_831957; top row) showing unique mutant variants with single-letter amino acid codes and color scheme for fitness at high TMP (yellow for fitness ≥ −1 and blue for fitness < −1). (**C**) Median fitness of *S. pneumoniae* DHFR homolog across the TMP gradient, with the reference sequence in red, TMP-resistant mutants in yellow, and nonresistant mutants in blue. (**D**) Grid plot of amino acid substitutions in *S. pneumoniae* (WP_002205327; top row), showing unique mutant variants and colors indicating fitness at high TMP (yellow for fitness ≥ −1 and blue for fitness < −1).

For *B. cereus*, numerous random mutant variants of the reference sequence were generated through our DropSynth assembly, revealing a range of fitness responses across the TMP gradient (Fig. 6A). Among these, only one mutant, featuring a valine-to-alanine substitution at position 71 (V71A), conferred resistance to TMP at all concentrations (Fig. 6B). This mutant exhibited increasing fitness across the gradient, whereas the reference sequence’s fitness declined (Fig. 6A).

For *S. pneumoniae*, we identified seven unique mutant variants conferring resistance at high TMP concentrations (Fig. 6C). These included four single-substitution variants (V48L, Q91R, P111S, and I142T), one double-substitution variant (K4N_Q107H), one triple-substitution variant (E36Q_L39T_N40G), and one quadruple-substitution mutant variant (H26Y_E36A_L39T_N40G) (Fig. 6D). The triple- and quadruple-substitution variants shared mutations at residues 36, 39, and 40, but the quadruple mutant variant (H26Y_E36A_L39T_N40G) showed higher fitness across the TMP gradient (Fig. 6C). The remaining 13 mutant variants recovered failed to restore metabolic function under high TMP selection (400× MIC), indicating that substitutions, including a single deletion (V77X), at these positions do not confer TMP resistance (Fig. 6D).

We recovered a perfect assembly *E. coli* DHFR homolog with a single–amino-acid substitution, lysine (K) to asparagine (N) at residue 38, which conferred increased resistance to TMP relative to the WT *E. coli* reference. This K38N variant showed improved resistance at higher TMP concentrations, with fitness values greater than −1 at 50 and 200 μg/ml (400× MIC), significantly outperforming the WT reference (see fig. S13). As a perfect assembly homolog, we recovered 1085 total barcodes for the K38N variant, including 169 high-confidence barcodes with at least 10 sequences per barcode, ensuring reliable fitness data. These results reveal the robust fitness of the K38N variant across the TMP gradient, with dial-out PCR validation underway to confirm its role in conferring resistance at high TMP concentrations.

We also analyzed fitness data for homologs from the most abundant phyla in our DropSynth-assembled library, including Bacillota, Bacteroidota, and numerous classes within Pseudomonadota (table S1). Across 224 species and their mutant variants, we identified 40 species with mutations conferring resistance to high TMP concentrations (200 μg/ml). Specifically, resistance was observed in 1 of 6 Alphaproteobacteria species, 1 of 17 Betaproteobacteria species, 7 of 58 Gammaproteobacteria species, 18 of 92 Bacillota species, and 13 of 51 Bacteroidota species (figs. S14 to S18). This diverse spread of resistant mutations underscores the adaptive capacity and evolutionary plasticity of the DHFR enzyme in response to antibiotic pressure. By generating a comprehensive, quantitative, and experimentally tractable dataset, this study provides a foundation for investigating resistance mutations, specific variants, and underlying sequence-function relationships. Furthermore, this framework enables systematic exploration of evolutionary and mechanistic questions across a broad sequence space, including homologs from unculturable taxa.

### Multiplexed fitness validation using dial-out PCR

Our final objective was to validate pooled assay results using dial-out PCR (*32*) to measure the growth rates of individual homologs across the TMP gradient. This validation step was necessary to confirm the accuracy of our high-throughput pooled assays and to ensure that the observed fitness effects were not artifacts of the competitive growth environment. Each DropSynth-assembled DHFR homolog is tagged with a unique barcode, enabling retrieval via PCR amplification. This barcoding system allows for precise identification and isolation of specific homologs from the pooled library, facilitating individual growth rate measurements (see Materials and Methods). We strategically targeted 10 DHFR homologs spanning a broad fitness range at TMP (200 μg/ml) (median log_2_ fold change: −9.1 to +6.2; table S10), ensuring representation of fitness profiles observed in the pooled assays. This selection included homologs from the pathogenic bacteria *B. cereus* and *S. pneumoniae* (table S11), which were of particular interest due to their clinical relevance and potential TMP resistance (Fig. 6). WT *E. coli* DHFR and mCherry served as positive and negative controls, respectively. Growth rates of dial-out homologs were compared to their fitness values from pooled assays across the TMP gradient (see fig. S19).

**Fig. 7.**
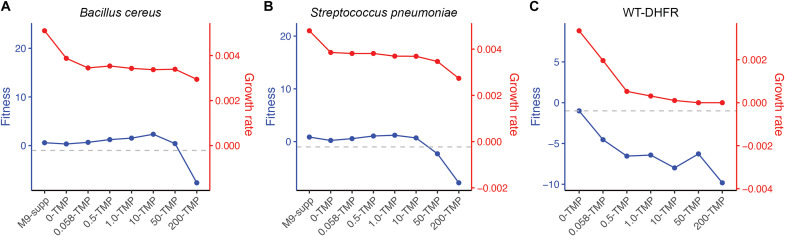
Dial-out validation for pathogenic DHFR homologs. Line plots showing experimentally determined fitness from pooled assays (blue) and growth rates of individual homologs measured via dial-out PCR and plate reader assays (red) for homologs of the pathogenic species (**A**) *B. cereus* and (**B**) *S. pneumoniae*, as well as the positive control (**C**) WT *E. coli* DHFR homolog. M9-supp, M-supplemented. Growth rates (per minute) were calculated as the maximum slope of optical density (OD)_600_ versus time on a log-linear plot.

For example, DHFR homologs from *B. cereus* and *S. pneumoniae* showed growth rate patterns across the TMP gradient that closely mirrored their fitness responses in the pooled assay (Fig. 7, A and B). Although these dial-out variants differed from the reference sequences used in the resistance analysis (Fig. 6), they followed similar trends and were well suited for validation (fig. S20). Their relatively stable growth rates up to TMP (50 μg/ml) aligned with consistent fitness values (≥−1), indicating inherent resistance at low to middle TMP levels. In contrast, the WT *E. coli* DHFR homolog showed progressively reduced growth across the gradient, consistent with its declining pooled fitness due to TMP inhibition (Fig. 7C). Spearman correlation analyses across all dial-out homologs confirmed significant positive correlations between growth rate and pooled fitness at multiple TMP concentrations (e.g., *r*_s_ = 0.67 and *P* = 0.028 at 0 μg/ml; *r*_s_ = 0.75 and *P* = 0.012 at 0.5 μg/ml; and *r*_s_ = 0.67 and *P* = 0.024 at 50 μg/ml) (fig. S21), providing independent validation of the pooled assay results.

In addition, a pilot experiment using the codon 1 library showed strong concordance in homolog fitness measurements with the main codon 1 dataset across the TMP gradient (Pearson *r*: 0.48 to 0.86 and *P* < 4.87 × 10^−39^ for all conditions; fig. S22), providing independent biological replication of our findings. The weakest correlation was observed at the highest TMP concentration (200 TMP; *r* = 0.48 and *P* = 4.87 × 10^−39^; fig. S22F), likely due to the limited dynamic range under this condition, where strong selection allowed growth of only a few highly resistant homologs. Despite this constraint, the overall consistency across conditions and between studies, combined with internal technical replication from barcode-tagged cells and independent dial-out validation, underscores the robustness, reproducibility, and reliability of our experimental framework.

## DISCUSSION

This study presents the most comprehensive analysis to date of homolog complementation and inhibitor tolerance within the DHFR protein family. Using a synthetic metagenomics approach, we applied DropSynth (*29*, *30*) to assemble 1208 unique DHFR homologs from two codon versions of a 1536-member library, enabling large-scale functional analysis of metagenome-derived sequences (Fig. 1). Combined with multiplexed functional assays, this allowed us to assess DHFR responses to TMP across a broad evolutionary range. Unlike traditional DMS (*14*–*16*), which focuses on single-protein variants, our approach captures natural sequence diversity and reveals a more nuanced view of fitness landscapes and resistance phenotypes. While not exhaustive, our findings highlight patterns of mutational constraint and resistance that can inform future studies of DHFR evolution and support efforts to predict resistance mechanisms and guide antibiotic design in diverse pathogenic bacteria.

### Insights into sequence-function relationships in DHFR protein evolution

To address our first objective, we conducted a complementation assay to evaluate the functionality of our assembled DHFR libraries in an *E. coli* knockout strain. The results demonstrated functional conservation of DHFR across diverse species despite significant sequence divergence. Notably, more than half of all perfect assembly homologs successfully rescued the *E. coli* ∆*folA* phenotype, underscoring the enzyme’s evolutionary resilience (Fig. 2B). Furthermore, when we incorporated mutant variants with up to five amino acid changes, the rescue rate increased to more than 90% (Fig. 2C). This substantial improvement in complementation efficiency highlights the adaptability of DHFR function through minor amino acid alterations, revealing the protein’s capacity to maintain its essential role despite sequence variations. However, we observed variations in complementation efficiency between codon versions for many homologs, suggesting that even subtle sequence differences can substantially affect protein functionality (Fig. 2D). These findings underscore the complex interplay between sequence and function in the DHFR protein family and highlight the importance of considering both amino acid composition and codon usage when studying protein evolution and functionality in heterologous expression systems (*45*, *46*).

To assess codon-specific effects, we used the Ribosome Binding Site (RBS) Calculator (version 2.1) (*47*) to predict translation initiation rates (TIRs) for 100 matched homologs present in both codon libraries. No significant difference in mean TIR was observed between codon versions (*t* test = 0.615 and *P* = 0.540), indicating no systematic bias. However, 60% of homologs showed ≥50% change in predicted TIR, and 54% differed by twofold or more, highlighting strong protein-specific effects. DHFR functions within a narrow expression “Goldilocks zone” where both under- and overexpression can impair growth (*37*), and multiple factors beyond catalytic activity likely shape fitness outcomes. These include protein stability and folding (influenced by chaperones and proteases) (*23*, *48*), metabolic coupling with thymidylate synthase (*40*, *49*), and protein-protein interactions (*37*). Together, these findings illustrate the complex relationship between sequence, structure, and function in protein evolution and emphasize the protein-specific impact of synonymous codon choice, even without global bias.

Using BMS, an innovative approach for mapping the fitness landscape of protein homologs generated through large-scale gene synthesis, we gained key insights into the mutational landscape of DHFR homologs (Fig. 3). First, conserved sites exhibited significantly constrained mutational fitness, emphasizing their critical role in protein function, with a strong negative correlation (ρ = −0.51), indicating that mutations at these positions likely disrupt stability or function (fig. S4A). Second, buried residues had smaller variability in fitness than solvent-accessible residues, possibly due to the potential impact of mutating buried residues on structure (fig. S4B). Third, the correlation between higher mutational coverage and increased positional fitness suggests that highly deleterious mutations are less likely to be captured by the assay due to the loss of the variant or insufficient reads to pass quality filters (fig. S4C). This effect may introduce a bias toward higher fitness values, as low-fitness homologs were excluded. Last, the lack of significant differences in BMS fitness across secondary structures highlights the complexity of mutational effects while also underscoring the potential variability in structural features among homologs (fig. S4D). Projecting homologs onto the *E. coli* reference assumes structural conservation across the protein family, which may not fully account for such differences.

Our GOF analysis further revealed how specific mutations can rescue metabolic function under complementation conditions. For example, we identified nearly 500 GOF mutants with single–amino-acid substitutions associated with 191 dropout homologs (Fig. 3A). Mapping these mutations to the WT *E. coli* DHFR sequence (Fig. 3E) revealed key residues in the NADPH binding pocket, including positions 97 and 98, which form hydrogen bonds with the nicotinamide ring to aid catalytic positioning (*50*), and position 17 in the Met20 loop, which stabilizes substrate and cofactor binding (*44*, *51*). GOF mutations included both buried and surface-exposed residues, emphasizing the structural and functional importance of these sites in fitness recovery and their potential for evolutionary adaptation and therapeutic targeting.

### Complex mechanisms underlying TMP resistance across the DHFR protein family

For our second objective, the TMP resistance assays revealed a spectrum of antibiotic susceptibility among DHFR variants, indicating that resistance operates along a continuum rather than as a binary trait (Fig. 4). Some variants exhibited moderate resistance, while others displayed high levels, suggesting that the degree of resistance is influenced by multiple factors, such as specific mutations in the DHFR gene, changes in enzyme activity, and alterations in substrate binding. These findings align with established mechanisms of TMP resistance, such as competitive binding of TMP to the folate pocket and the role of active-site substitutions in conferring resistance (*11*, *52*, *53*). For example, the decline in the number of resistant homologs with increasing TMP concentration reflects the progressive loss of DHFR function under TMP pressure (Fig. 4D), highlighting the complexity of resistance mechanisms, as amino acid substitutions become less viable at higher drug levels (Fig. 5D). This spectrum challenges simplistic models of resistance and highlights the need for more sophisticated approaches in antibiotic development, such as targeting multiple variants or developing compounds that inhibit both WT and resistant forms (*54*, *55*).

The maintenance of functionality in a small subset of homologs at high TMP concentrations suggests the presence of resistance-conferring mutations. This aligns with previous findings that identify specific residue variations, such as D27E and L28Q, as common structural elements in TMP-resistant *E. coli* DHFR variants (*11*, *56*). Furthermore, the fitness differences between closely related and distantly related homologs under TMP selection support the idea that resistance often emerges through specific mutations in the DHFR gene, with evolutionary proximity influencing the likelihood of sharing resistance-conferring mutations (*57*). Here, the relationship between phylogenetic relatedness and fitness responses among DHFR homologs proved complex. While some closely related homologs exhibited similar fitness profiles (Fig. 5A), others showed unexpected divergence, especially as the selective pressure of TMP increased across the experimental gradient (Fig. 5B). This variability highlights the intricate nature of protein evolution and the challenges of predicting functional outcomes based solely on sequence similarity. These findings have substantial implications for both clinical practice and evolutionary biology, emphasizing the importance of species-specific antibiotic selection in clinical settings and enhancing our understanding of resistance as an evolutionary adaptation.

### Identifying key resistance-conferring mutations in DHFR homologs of pathogenic bacteria

In our third objective, we identified mutations that confer TMP resistance in DHFR homologs from pathogenic bacterial species, including members from the ESKAPE group (*34*, *35*). This approach revealed key resistance-associated substitutions in clinically relevant pathogens, such as V71A in *B. cereus* and V48L, Q91R, P111S, I142T, and multisubstitution variants in *S. pneumoniae* (Fig. 6). Notably, we identified the K38N mutation in *E. coli* DHFR, which conferred increased resistance at the highest TMP concentrations tested (fig. S13). While a previous deep mutational scan of WT *E. coli* DHFR under mild TMP pressure (3 μg/ml) also detected N38 variants, they appeared at much lower frequency than the WT K38 (0.14% versus 99.57%) (*58*). Our data suggest that N38 provides a resistance advantage primarily at higher TMP concentrations, indicating a concentration-dependent effect. These findings underscore how single–amino-acid substitutions can substantially alter drug susceptibility, highlighting the need to monitor even subtle genetic variation in clinical settings.

### Validation of high-throughput fitness measurements

For our final objective, we validated our pooled assay results using dial-out PCR (*32*) to measure individual growth rates of selected DHFR homologs across the TMP gradient, providing critical support for the accuracy and reliability of our high-throughput approach. We selected 10 homologs spanning a broad fitness range, including several from clinically relevant pathogenic bacteria, to assess whether fitness effects observed in the pooled assay reflected true biological performance rather than artifacts of competitive growth or compositional bias. The strong concordance between individual growth rates and pooled fitness responses, particularly for homologs from pathogenic *B. cereus* and *S. pneumoniae* (Fig. 7), underscores the robustness of our experimental design. Spearman’s correlation analysis further revealed significant positive relationships between dial-out growth rates and pooled fitness across TMP concentrations (fig. S21), independently validating the multiplexed assay results. These findings confirm the reliability of our pooled fitness measurements and reinforce the broader conclusions about the DHFR fitness landscape in response to TMP stress.

### Power of DropSynth and future directions

Our synthetic metagenomics approach, while similar in concept to DMS, focuses on naturally occurring homologs from diverse species rather than variants of a single protein, enabling broader exploration of evolutionary diversity and functional conservation within the DHFR family. By integrating high-throughput, multiplexed gene synthesis with pooled functional assays, we overcome longstanding barriers to studying diverse protein families and reveal how DHFR enzymes evolve, adapt, and acquire antibiotic resistance. Examining a wide range of metagenome-derived variants provides deeper insights into essential structural and catalytic features, advancing our understanding of DHFR function across species and physiological contexts, with broad implications for protein engineering, drug development, and molecular evolution.

Our findings also have notable implications for ML models in protein function prediction and generative models, particularly given that most current models rely solely on uncharacterized metagenomic sequences in an unsupervised manner (*59*, *60*). Our observation that nearly half of all metagenome-derived homologs failed to complement effectively highlights the importance of thorough functional characterization and underscores a well-documented challenge with the heterologous expression of proteins. Including noncomplementing sequences in training datasets without adequate validation could introduce noise or bias, potentially poisoning the training set and compromising model accuracy (*61*, *62*). This issue warrants further quantitative investigation, which will be a focus of our future work, to enhance the reliability of ML-based predictions in protein function and antibiotic resistance. Moreover, the observation that GOF mutations were present in nearly all noncomplementing homologs offers a compelling opportunity for ML models to uncover the underlying patterns and mechanisms driving functionality.

Looking ahead, we plan to extend this synthetic metagenomics approach to other essential bacterial enzymes and scale up to larger libraries capable of assembling over 10,000 homologs and millions of mutant variants in parallel. Future efforts will focus on developing advanced modeling techniques to improve the prediction of antibiotic resistance from sequence data, including methods less dependent on traditional sequence alignment. As we scale to more complex mutant libraries, accurately interpreting the effects of multiple co-occurring mutations will require careful consideration of epistatic interactions, as the phenotypes of multimutant variants often reflect nonadditive interactions that cannot be inferred from single-mutation effects alone. The successful application of DropSynth and BMS in this proof-of-concept study demonstrates the power of this platform for exploring resistance mechanisms across diverse protein families and lays the foundation for improved antibiotic development and therapeutic strategies.

## MATERIALS AND METHODS

### DropSynth library assembly

The methods for assembling the DropSynth libraries were previously described in detail by Sidore *et al.* (*30*). Briefly, the process involves five major components: (i) oligo design, where a pool of 33,792 230-mer oligos was designed and synthesized, encoding two codon versions of a 1536-member DHFR homolog library; (ii) barcoded bead design and assembly, which involved creating unique 12-mer barcodes and assembling them onto magnetic beads; (iii) oligo amplification and barcoding, where oligo subpools were amplified, processed, and hybridized to the barcoded beads; (iv) emulsion PCR assembly, during which the loaded beads were compartmentalized in droplets and underwent assembly via PCR; and (v) bulk suppression PCR, which involved amplifying the assembled gene libraries using specific primers and conditions to obtain the final product. See Fig. 1A for a conceptual overview.

### pCVR205 plasmid construction

The DHFR expression vector for *E. coli* was derived from plasmid SMT205 (Addgene #134817), which supports leaky expression with lac operon (lac-o) repression, eliminating the need for isopropyl-β-d-thiogalactopyranoside induction to avoid toxic DHFR accumulation (*33*). The plasmid was modified by removing unnecessary restriction sites originally used for cloning and reintroducing three sites compatible with the *folA* gene library (see fig. S23 for reference). NdeI and KpnI sites were removed from the WT *thyA* gene by leveraging genetic code degeneracy, and a gBlock was designed to insert one KpnI site and one BspQI site at the 3′ end of the DHFR gene. This gBlock was integrated into the SMT205 backbone using Golden Gate assembly, creating the plasmid pCVR1. The gBlock insertion was validated by Sanger sequencing using internal and external primers to the *thyA* region. Site-directed mutagenesis was then used to introduce an NdeI site at the 5′ end of *folA*, resulting in the final construct, pCVR205. The plasmid was transformed into *E. coli* DH5α cells, and colonies were confirmed through Sanger sequencing. The final plasmid is available on Addgene (#198715). The gBlock sequence and primers used are provided in the Supplementary Materials (table S12).

### Ligation of barcoded libraries into the pCVR205 plasmid

DHFR libraries were ligated to the digested pCVR205 plasmid at a 3:1 insert-to-vector molar ratio, then purified, and eluted. Approximately 170 ng of the ligated product from each library was transformed into 25 μl of DH5α cells, which were allowed to recover in super optimal broth (SOB) medium before plating on LB agar with 10× dilutions to estimate colony numbers. Each library was initially bottlenecked at ~1 million colony-forming units (CFUs) during transformation into the DH5α cloning strain. The libraries were recovered by scraping the cells from the plates, pelleting them, and extracting plasmid DNA using the Monarch Plasmid DNA Miniprep Kit (New England Biolabs).

### Assembly barcode sequencing and annotation

The assembly barcoded libraries were sequenced using five paired-end 600-cycle runs on an Illumina MiSeq. After PCR amplification with sequencing primers, amplicons were gel extracted, quantified using an Agilent 2200 TapeStation, and pooled for sequencing with custom MiSeq primers. All primers are available in our previous study (*30*). Individual FASTQ files were downsampled to 1,880,288 reads to address coverage biases, and adapter sequences were trimmed with BBDuk (*63*). Paired-end reads were merged using BBMerge (*63*), and a custom Python script generated consensus nucleotide sequences for each barcode, mapping them to their corresponding variants. Barcodes linked to multiple variants were filtered on the basis of Levenshtein distance to identify contamination (*64*). A consensus sequence was generated from the majority base call, and variants were imported into R (*65*) for coverage and fidelity analysis. Assemblies represented refer to the number of assemblies corresponding to a perfect amino acid sequence, while percent perfect assemblies indicate the median percentage of perfect sequences from constructs with at least 100 assembly barcodes. Mutant homolog sequences were annotated by aligning the consensus nucleotide sequence against designed DHFR homologs, extracting the closest matches, and performing pairwise amino acid alignments to annotate mutations within five amino acids of the designed sequence.

### DHFR library transformation into folate-deficient *E. coli* knockout model

DHFR is an essential gene that converts 7,8-dihydrofolate (DHF) to 5,6,7,8-tetrahydrofolate, a key step in deoxythymidine phosphate biosynthesis (see fig. S24). For the complementation assay, barcoded DHFR libraries were transformed into the folate-deficient *E. coli* knockout strain *ER2566* Δ*folAΔthyA* (*33*), which requires external folate and thymidine for growth. Controls included the WT *E. coli* DHFR variant, the low-function D27N variant (*38*), and mCherry, a noncatalytic red fluorescent protein (*39*). Control plasmids were barcoded with 20 randomized nucleotides via In-Fusion HD cloning. Fifteen colonies from each control were sequenced and mixed with the library at a 0.1% molar ratio.

The combined library and controls (50 ng in total) were transformed in quadruplicate into 80 μl of *ER2566 ΔfolAΔthyA* cells by electroporation. After recovery in SOB medium, cells were plated on LB agar containing chloramphenicol and thymidine and incubated at 37°C for 22 hours. The resulting colonies were scraped and resuspended in supplemented M9 medium (80 μM adenosine, 0.5 mM glycine, 4.5 mM inosine, 4.2 μM calcium pantothenate, 0.5 mM methionine, and 0.21 mM thymidine), diluted to an optical density (OD)_600_ of 3, and plated again on the same medium for an additional 22-hour growth period. Transformation efficiency was quantified by plating six 10-fold serial dilutions on supplemented M9 medium and incubating for 22 hours at 37°C. This procedure yielded 14 million CFUs for the codon 1 library and 44 million CFUs for codon 2. Growth and selections were subsequently conducted using populations exceeding 1 billion cells per condition, ensuring robust coverage of library diversity and minimal sampling error throughout the experiment.

### Complementation assay

For the complementation assay, cells from the initial transformation on supplemented M9 medium plates were scraped, washed via three centrifugation rounds at 2500 relative centrifugal force (rcf) for 5 min, and resuspended in minimal M9 medium [M9 salts, 0.4% glucose, 2 mM magnesium sulfate, and chloramphenicol (35 μg/ml)] to an OD_600_ of 3 U/ml. Stocks were prepared at a 1:1 ratio of cells to 50% glycerol and stored at −80°C. Five milliliters of the washed cells were plated on minimal M9 medium and incubated at 37°C for 22 hours to evaluate the ability of DHFR variants to restore metabolic function in the *E. coli ER2566* ∆*folA∆thyA* knockout strain without any supplementation. Resulting colonies were scraped, resuspended in minimal M9 medium, and miniprepped using a Monarch Plasmid DNA Miniprep Kit (New England Biolabs) for plasmid extraction and sequencing. This process was repeated for four rounds, with colonies from each round replated to initiate the next. However, only first-round results were analyzed because of challenges in normalizing barcode recoveries across time points.

### TMP selection gradient

A TMP gradient ranging from 0 to 400 times the MIC of 0.5 μg/ml (*66*) was prepared in minimal M9 medium, with concentrations of 0 μg/ml (complementation), 0.058 μg/ml, 0.5 μg/ml (MIC), 1.0 μg/ml, 10 μg/ml, 50 μg/ml, and 200 μg/ml (400× MIC). Washed and diluted cells (OD_600_ of 3 U/ml) were spread evenly on agar plates with the TMP gradient and incubated at 37°C for 22 hours to evaluate the resistance of DHFR variants to the antibiotic. Resulting colonies were scraped, resuspended in minimal M9 medium, and miniprepped using a Monarch Plasmid DNA Miniprep Kit (New England Biolabs) for plasmid extraction and sequencing. As with complementation, this process was repeated for four rounds of TMP treatment, with colonies from each round replated at the same TMP concentration to initiate the next round. Only first-round results were analyzed because of challenges in normalizing barcode recoveries across time points.

### Barcode sequence screening

Plasmid barcodes recovered from all nine sampling conditions were independently amplified using unique primer sets: C1: LB medium with chloramphenicol and thymidine, C2: minimal M9 medium with supplements, C3: minimal M9 medium without supplements (complementation), C4: TMP (0.058 μg/ml), C5: TMP (0.5 μg/ml; MIC), C6: TMP (1 μg/ml), C7: TMP (10 μg/ml), C8: TMP (50 μg/ml), and C9: TMP (200 μg/ml; 400× MIC). PCR was performed to amplify plasmid barcodes using five primer pairs that introduced custom sequencing adapters and library indexes compatible with Illumina sequencing. Plasmid primer sets and sequencing primer pairs are provided in the Supplementary Materials (table S13). The resulting amplicons were size selected via gel extraction, purified, pooled, and sequenced on an Illumina NovaSeq 6000 S4 (35-cycle run), generating 1,010,919,383 total sequence reads across both codon version libraries (table S2). Barcodes for each sampling condition were clustered using Starcode (*67*) to collapse barcodes within a Levenshtein distance of 1 (*64*).

### Fitness calculations

To reduce noise in calculating fitness changes between sampling conditions, we applied a two-tier filtering strategy. First, we retained only barcodes with ≥10 reads under at least one experimental condition to minimize the impact of low-abundance noise. Second, we included only homologs represented by at least five of these high-confidence barcodes to ensure reliable internal replication. This filtering reduced the total number of unique barcodes from 474,789 to 323,256, with 712,211,978 reads successfully mapped across both codon version libraries, representing ~70% of all sequenced reads (table S2). Fitness scores were calculated for each mapped variant associated with at least five high-confidence barcodes. To account for variation in sequencing depth, read counts were normalized within each condition relative to the total number of reads recovered under condition C2 (growth in M9 minimal medium with supplements). The log_2_ fold change in normalized barcode abundance between each test condition (C3 to C9) and the C2 baseline was then calculated using the following equation

$$\int_{0}^{\chi }={\text{log}}_{2}\left(r_{\chi }+1\right)-{\text{log}}_{2}\left(r_{0}+1\right)$$ (1)

where $r_{\chi }$ is the number of normalized reads under the corresponding sampling condition (C3 to C9) and $r_{0}$ is the number of normalized reads under sampling condition C2. We then took the median value (to minimize effects of outliers) of the log_2_ fold change for each unique DHFR variant based on its associated barcodes (BC) for each sampling condition using the following equation

$$\int_{0}^{\chi }{\text{seq}}_{A}=\text{median}\left(\int_{0}^{\chi }{\text{BC}}_{1A},\int_{0}^{\chi }{\text{BC}}_{2A},\int_{0}^{\chi }{\text{BC}}_{3A},\ldots \right.$$ (2A)

$$\int_{0}^{\chi }{\text{seq}}_{B}=\text{median}\left(\int_{0}^{\chi }{\text{BC}}_{1B},\int_{0}^{\chi }{\text{BC}}_{2B},\int_{0}^{\chi }{\text{BC}}_{3B},\ldots \right.$$ (2B)

### Broad mutational scanning

BMS was conducted on all homologs obtained from complementation (C3) and only those considered capable of complementation (median fitness > −1 at C3) across all TMP selection conditions (C4 to C9). We aligned all homologous sequences with Multiple Alignment using Fast Fourier Transform (MAFFT) and generated a residue-level lookup table. For each perfect assembly homolog sequence (mutations = 0), we scanned all residues and recorded their fitness in a BMS data table, linking each residue to its corresponding *E. coli* position based on the alignment. For mutant variants within five amino acids of the designed homolog, we documented only the mutated residues and added their fitness to the BMS table. The BMS fitness for each residue was calculated as the median of all corresponding data points at that position.

### GOF mutations

To identify GOF mutations in the complementation assay, we classified DHFR homologs with fitness scores below −1 as dropout homologs and selected them for further analysis. These dropout homologs were screened for single–amino-acid mutant variants that restored function (fitness > −1), designating these as GOF mutants. We aligned the sequences of the GOF mutants to the WT *E. coli* DHFR sequence, counted the number of mutations at each position, and identified positions as significant if the mutation count exceeded two SDs above the mean. These significant GOF positions were mapped onto the *E. coli* DHFR protein structure (PDB 4KJK) (*43*) to visualize their spatial and functional relevance. Last, we examined specific types of amino acid changes, including deletions, that contributed to fitness restoration at each significant position.

### DHFR fitness validation by dial-out PCR

Each DHFR assembly is tagged with a unique barcode, enabling the retrieval of specific homologs from each codon version library through PCR amplification, referred to as dial-out PCR (*32*). The dial-out procedure aims to independently verify an individual homolog’s fitness response to TMP in isolation, without competition effects from the pooled multiplex assay. The WT *E. coli* DHFR gene (NP_414590) served as a positive control, while mCherry was our negative control. Dial-out PCR primers were designed to flank each homolog construct, with reverse primers annealing to the gene-specific barcode (table S14). After gel extraction, individual amplicons were subjected to restriction digest using high fidelity (HF) restriction ezymes KpnI-HF and NdeI (New England Biolabs), ligated into an empty pCVR205 plasmid, transformed into electrocompetent *E. coli ER2566* ∆*folA∆thyA* cells, and incubated in M9 minimal medium with or without TMP on 96-well plates. OD_600_ measurements were recorded every 6 min for 22 hours using a Synergy Neo2 microplate reader (Agilent BioTek) to calculate growth rates.

### Statistical analysis

Data analysis and statistical tests, including Pearson’s (ρ) and Spearman’s (*r*_s_) correlations, Wilcoxon rank sum tests, and Welch’s *t* tests, were performed in R (version 4.3.2) (*65*). Data visualizations were generated using the ggplot2 (*68*) and ggtree (*69*) packages, while structural visualizations were created with UCSF Chimera (*70*). Residue conservation was assessed via Jensen-Shannon divergence (*71*), with secondary structure and relative solvent accessibility derived from DSSP analysis (*72*, *73*) of the 1H1T structure (*74*). Statistical significance was set at *P* ≤ 0.05.
