# Supplementary material for: Exploring antibiotic resistance in diverse homologs of the dihydrofolate reductase protein family through broad mutational scanning
Source: Sci Adv. 2025 Aug 13;11(33):eadw9178. doi: 10.1126/sciadv.adw9178 (PMC12346277; doi:10.1126/sciadv.adw9178)
Supplement: Supplementary file 1 — Figs. S1 to S24 Tables S1 to S14 [file sciadv.adw9178_sm.pdf]

Science Advances
AAAS

Supplementary Materials for

Exploring antibiotic resistance in diverse homologs of the dihydrofolate reductase protein family through broad mutational scanning

Karl J. Romanowicz *et al.*

Corresponding author: Calin Plesa, calin@uoregon.edu

Sci. Adv. **11**, eadw9178 (2025)
DOI: 10.1126/sciadv.adw9178

This PDF file includes:

Figs. S1 to S24
Tables S1 to S14

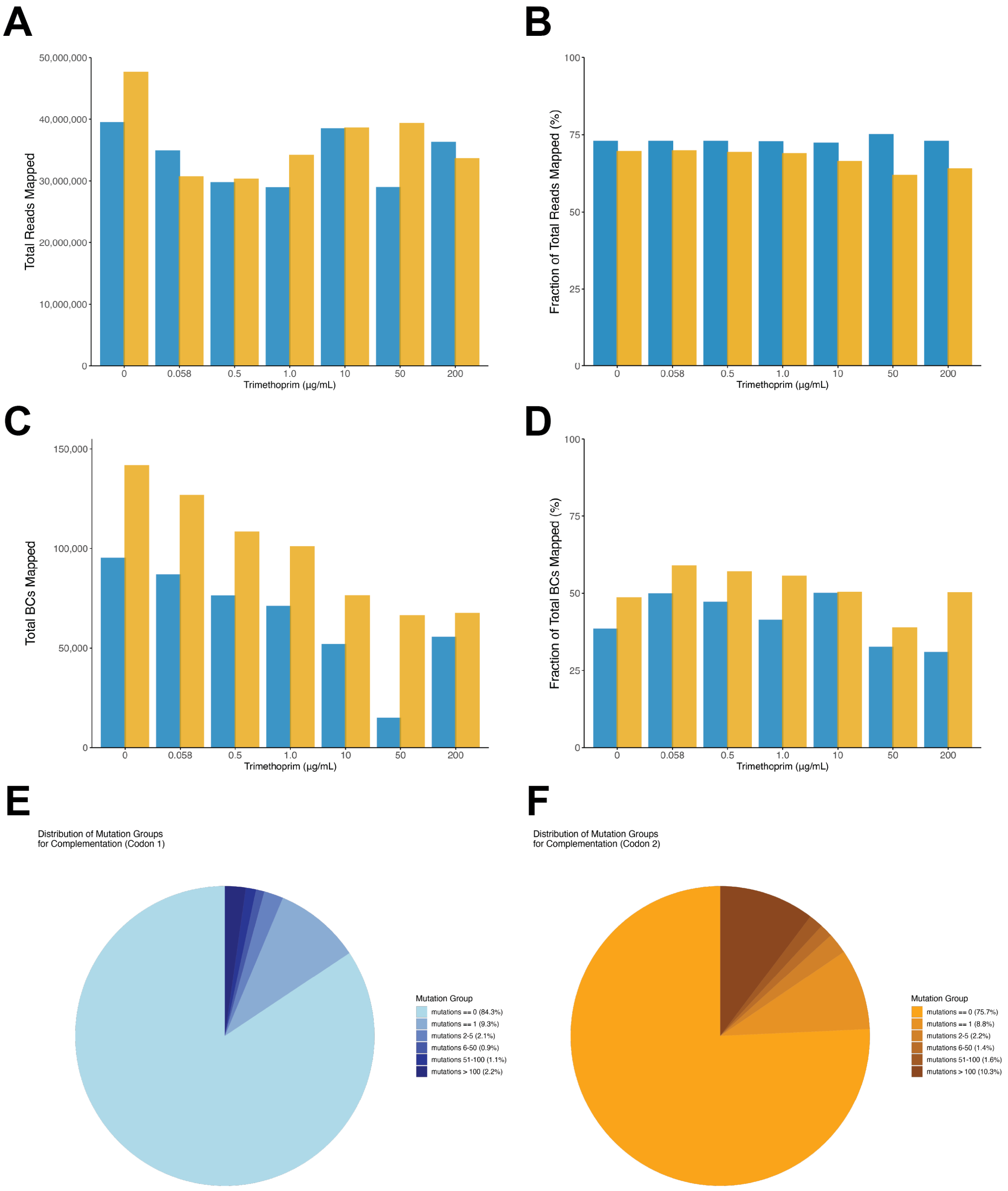

Fig. S1. Sequencing Statistics Across TMP Gradient. Sequencing reads and barcode statistics across the TMP gradient showing (**A**) total reads mapped at each TMP condition, (**B**) fraction of total reads mapped, (**C**) total barcodes (BCs) mapped, and (**D**) fraction of total BCs mapped. (**E**) The distribution of total reads mapped to barcodes by mutations in the Codon 1 library. (**F**) The distribution of total reads mapped to barcodes by mutations in the Codon 2 library.

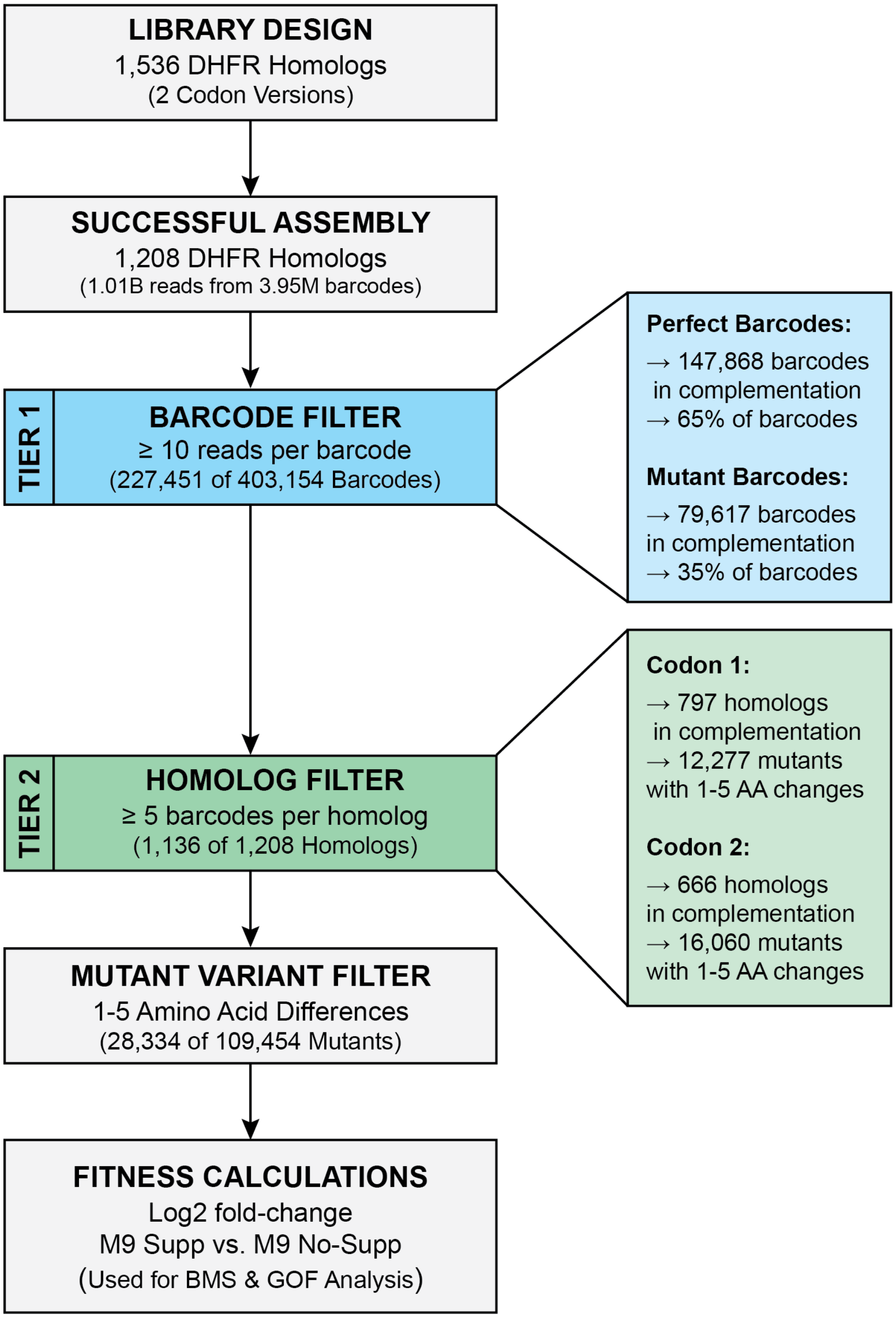

Fig. S2. Filtering and Retention Pipeline for Library Design and Analysis. Flowchart illustrating the progression from the initial DropSynth-designed DHFR library to the final sets of homologs and mutants used in complementation and mutational fitness analyses. Key filtering steps include removal of low-quality assemblies, mapping of barcodes to verified sequences, and application of a two-tier barcode filtering strategy: (1) retention of barcode sequences with ≥10 reads in at least one experimental condition, and (2) inclusion of only those homologs represented by ≥5 such high-confidence barcodes. This pipeline ensures robust replication and high-confidence variant inclusion in downstream analyses.

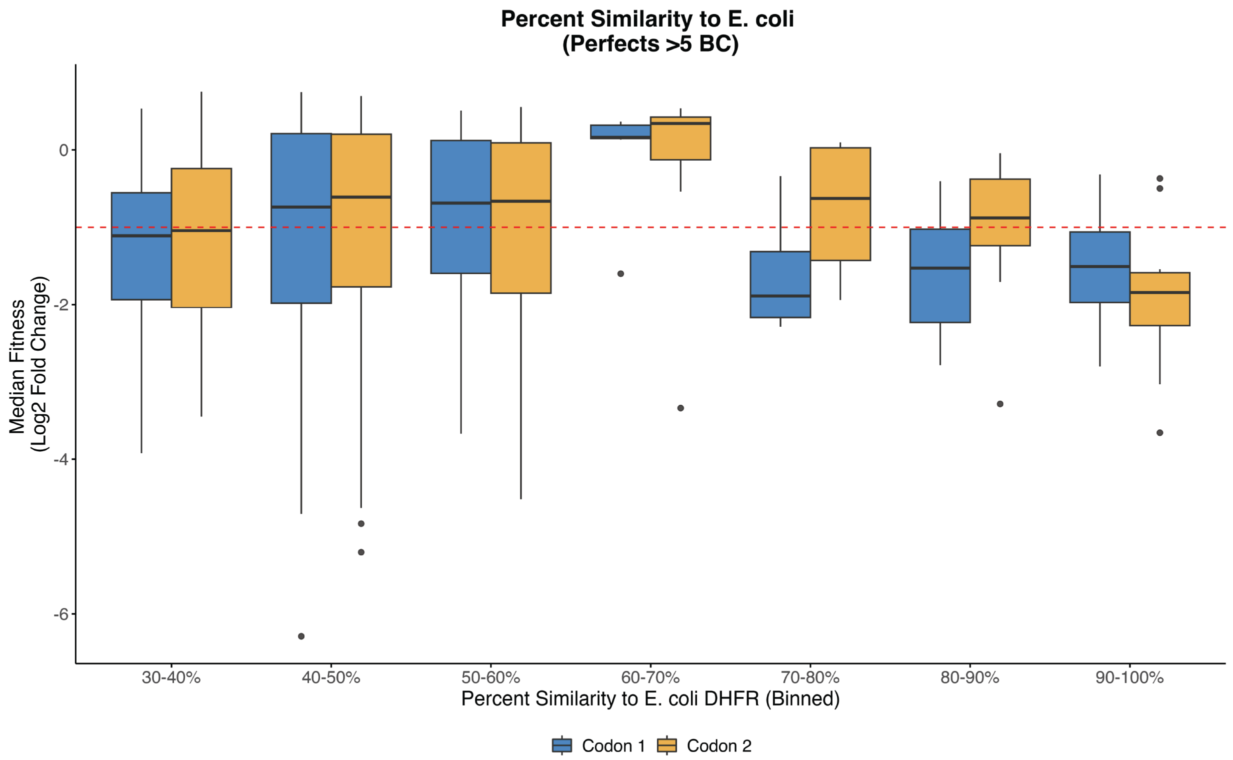

Fig. S3. Sequence Similarity of DHFR Homologs to Wild-Type *E. coli* DHFR. The median sequence identity of perfect assembly homologs recovered from the non-supplemented complementation condition was 46%. All DHFR homologs were grouped by percent similarity to the WT *E. coli* DHFR, with median fitness and standard deviation shown for each bin. Most distant homologs retained the ability to complement (fitness > -1) the function of native *E. coli* DHFR in the knockout strain. The red dashed line at -1 indicates the minimum fitness threshold for successful complementation.

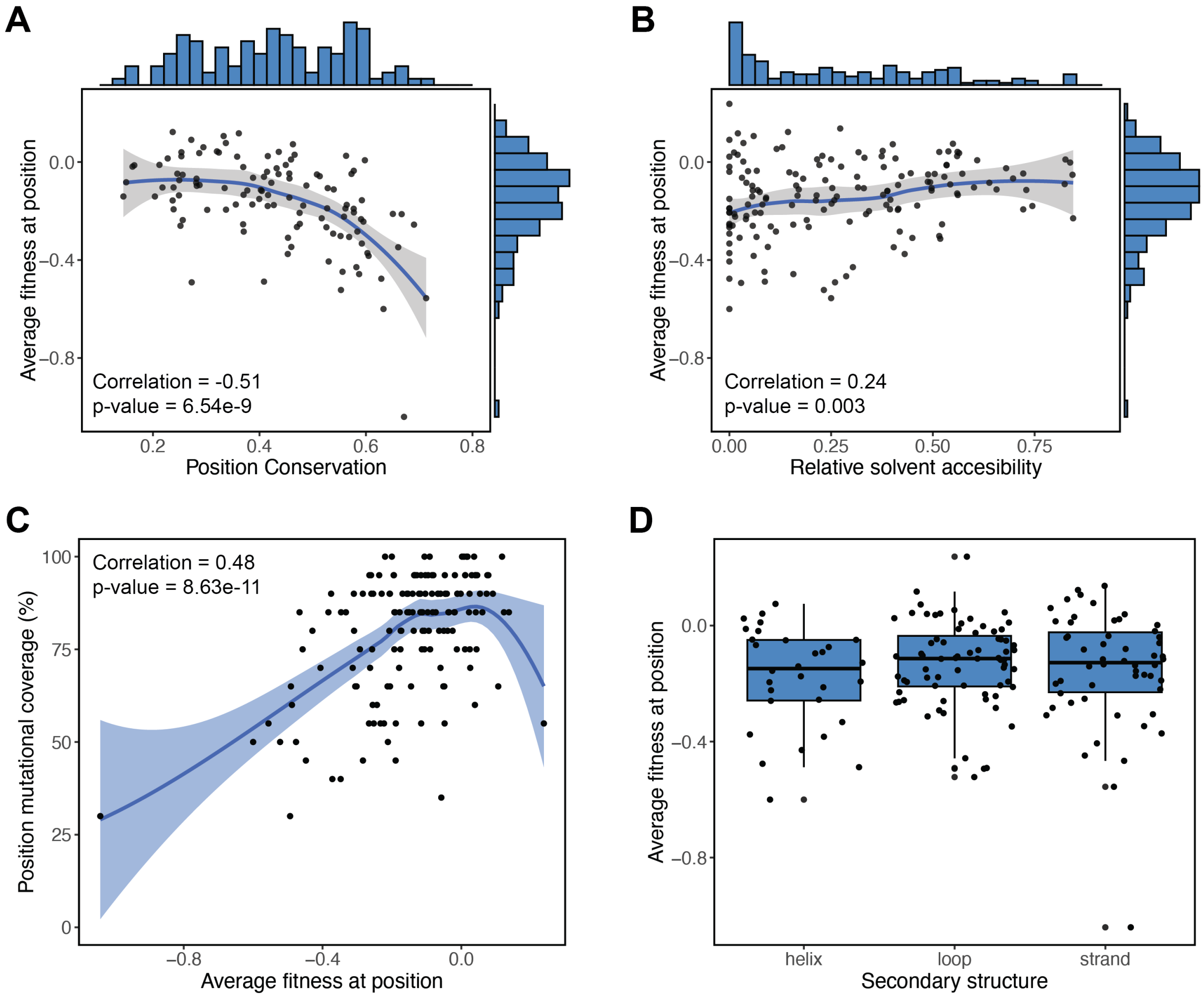

Fig. S4. Broad Mutational Scanning Summary Statistics at Complementation. (**A**) Average BMS position fitness compared to site conservation (Jensen-Shannon divergence) under complementation. (**B**) The average BMS position fitness compared to the relative solvent accessibility based on a DSSP analysis of the 1H1T crystal structure. Buried residues tend to be more constrained ($P = 0.003$). (**C**) Mutational scanning coverage decreases at sites of low fitness ($P = 8.63e^{-11}$) due to assembly barcodes with low read numbers. (**D**) Average BMS position fitness based on secondary structures in the E. *coli* DHFR structure (helix, loop, strand). No significant differences were observed (Kruskal–Wallis $P = 0.99$).

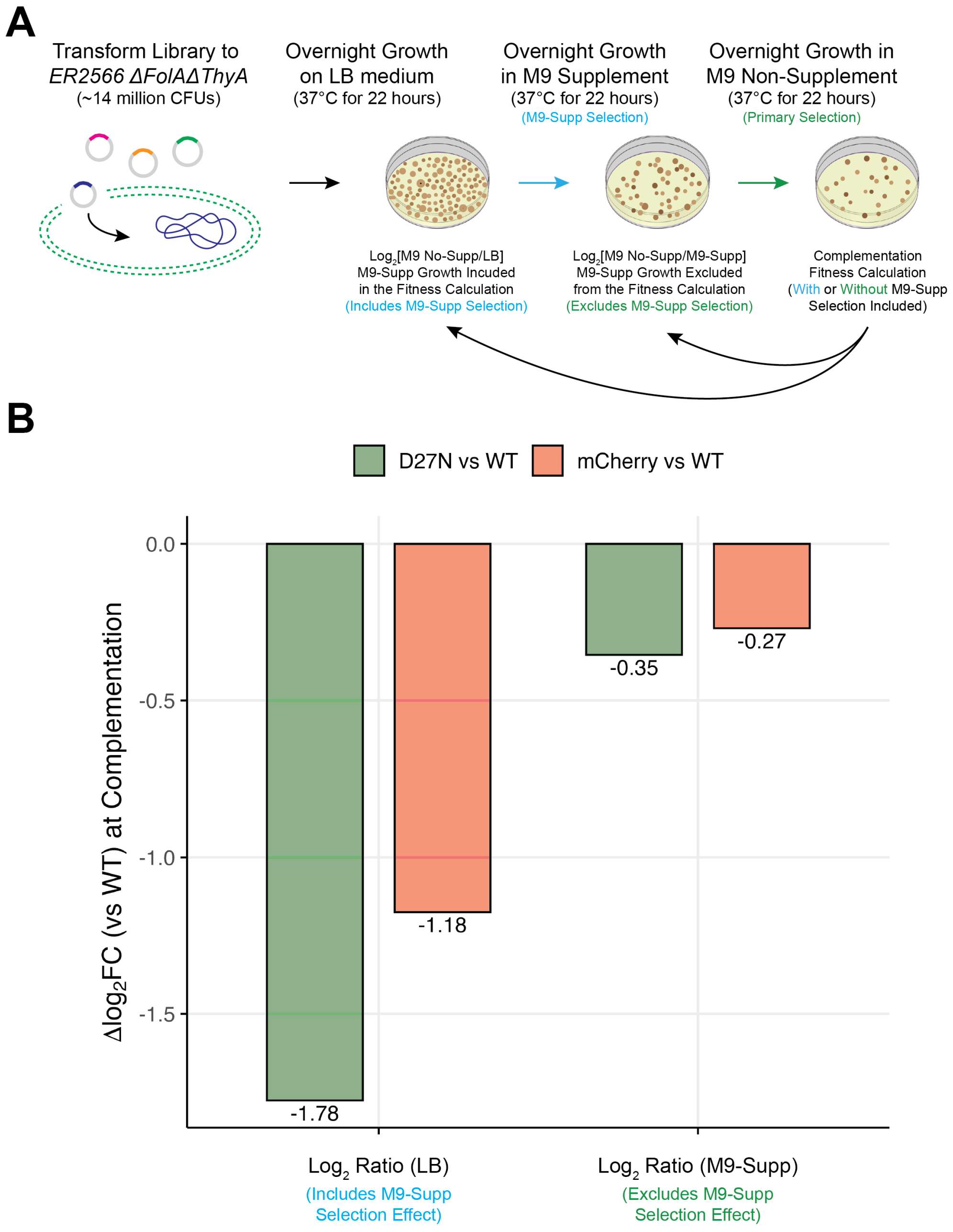

Fig. S5. Pre-selection in M9-Supplemented medium reduces the fitness gap between controls. (**A**) Timeline of DHFR-homolog library growth. ER2566 Δ*folA*Δ*thyA* cells were transformed and recovered overnight in LB, followed by deep sequencing ("LB"). Cultures were then washed and grown overnight in M9-Supplemented minimal medium before sequencing again ("M9-Supp"). Finally, cells were transferred to minimal M9 medium without TMP (complementation) or with the indicated TMP concentrations for overnight growth and final sequencing. Although intended as non-selective, the M9-Supplemented step imposed detectable selection pressure. Depending on whether the LB or M9-Supp sample is used as the denominator in $\log_2$ fold-change ($\log_2$FC) calculations, this intermediate selection step can be included or excluded. (**B**) Pre-selection effect on apparent fitness at 0 µg/mL TMP. Bars show the difference in mean $\log_2$FC between negative controls (D27N, mCherry) and the wild-type (WT) positive control, using either LB or M9-Supp as baseline. Values ($\Delta\log_2$FC = Negative Control − WT) indicate the relative separation between controls; smaller values reflect reduced fitness differences. Using M9-Supp as the baseline omits the selection already imposed in that condition, compressing the dynamic range of the complementation assay.

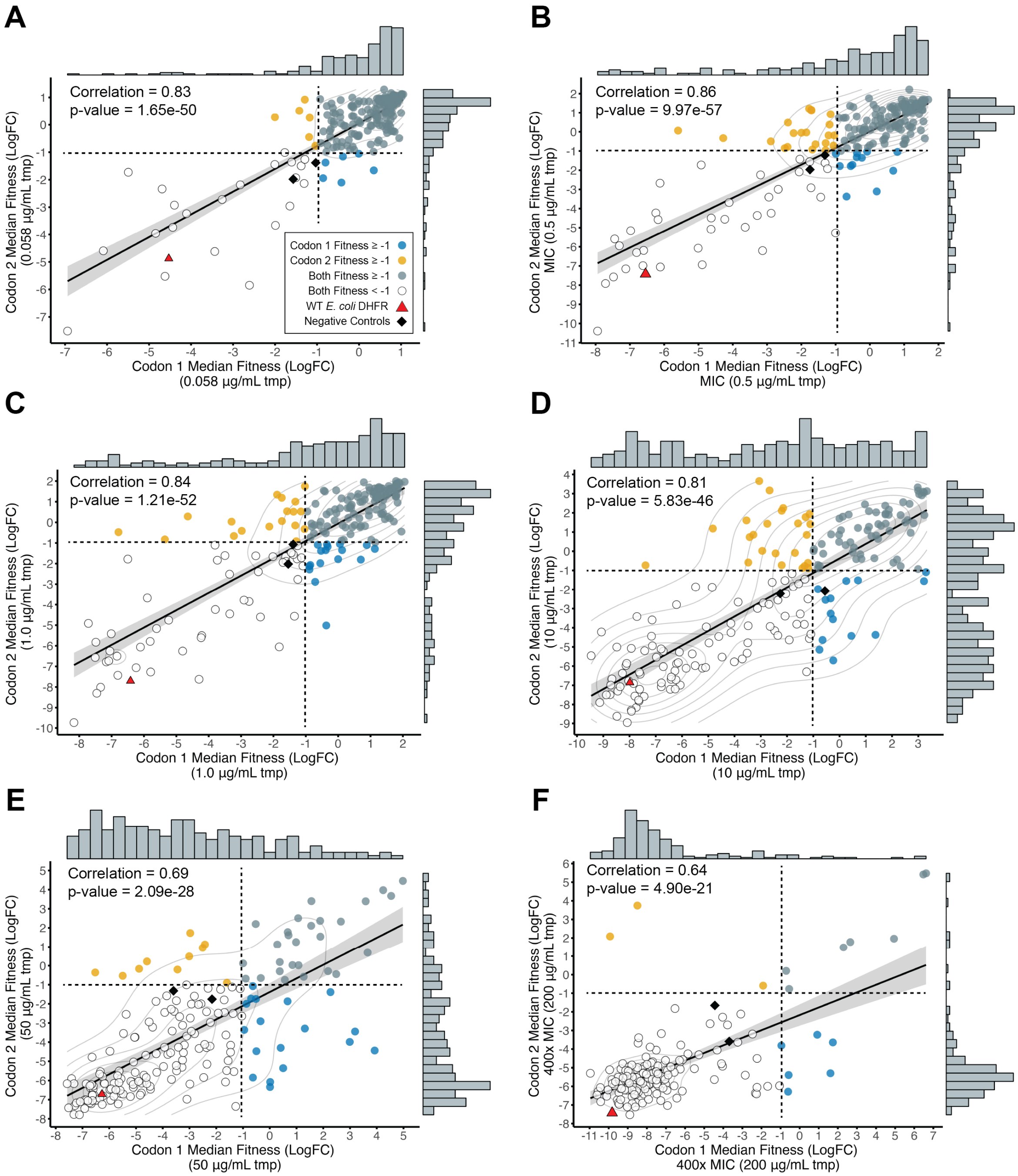

Fig. S6. Fitness Correlations for Homologs Shared Between Codon Versions Along the TMP Gradient. Pearson fitness correlations for shared homologs between codon versions at (**A**) 0.058 µg/mL TMP, (**B**) 0.5 µg/mL TMP (MIC), (**C**) 1.0 µg/mL TMP, (**D**) 10 µg/mL TMP, (**E**) 50 µg/mL TMP, and (**F**) 200 µg/mL TMP (400× MIC). Points are colored based on fitness for codon-specific homologs, shared homologs, or those with low fitness. The WT *E. coli* DHFR homolog and negative controls (D27N, mCherry) are plotted for reference. Dashed lines denote the minimum fitness thresholds for trimethoprim resistance.

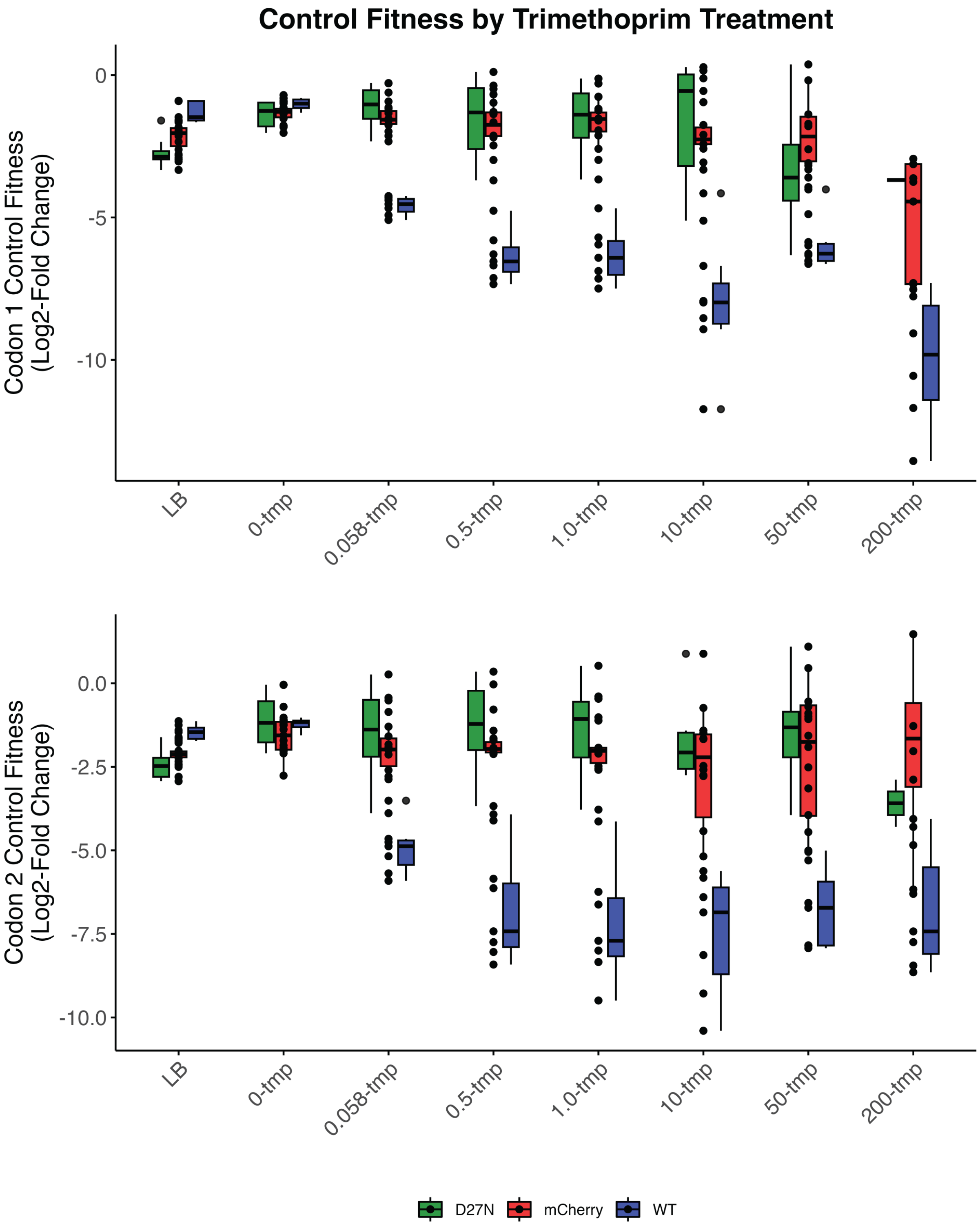

Fig. S7. Control Variant Fitness Under Trimethoprim. Boxplot illustrating homolog fitness across the trimethoprim gradient for Codon 1 (**top**) and Codon 2 (**bottom**), including the wild-type (WT) *E. coli* DHFR (positive control) and the negative controls, D27N and mCherry.

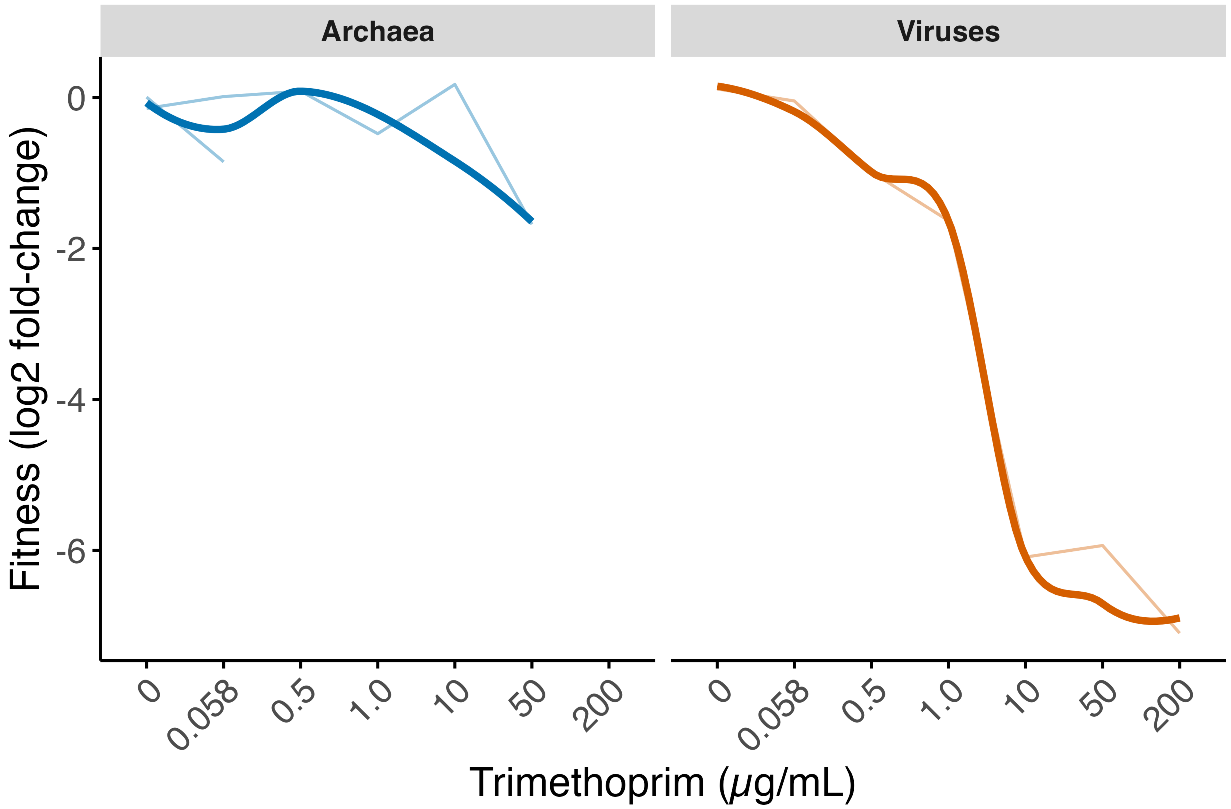

Fig. S8. Fitness response of archaeal and viral DHFR homologs across the trimethoprim gradient. Each line represents the fitness profile of an individual DHFR homolog from either an archaeal (left) or viral (right) taxon included in the Codon 1 dataset. Fitness was measured as log_2 fold-change relative to the M9-supplemented condition and plotted across seven TMP concentrations (0 to 200 μg/mL). While most homologs from these divergent taxa exhibited limited or no complementation in E. coli, a subset were capable of complementation (fitness > -1) and showed measurable growth and characteristic TMP-dependent declines in fitness. A smoothed average line is overlaid for each group to illustrate overall trends. These profiles highlight the rare but notable functional compatibility of distantly related DHFRs within the E. coli cellular context.

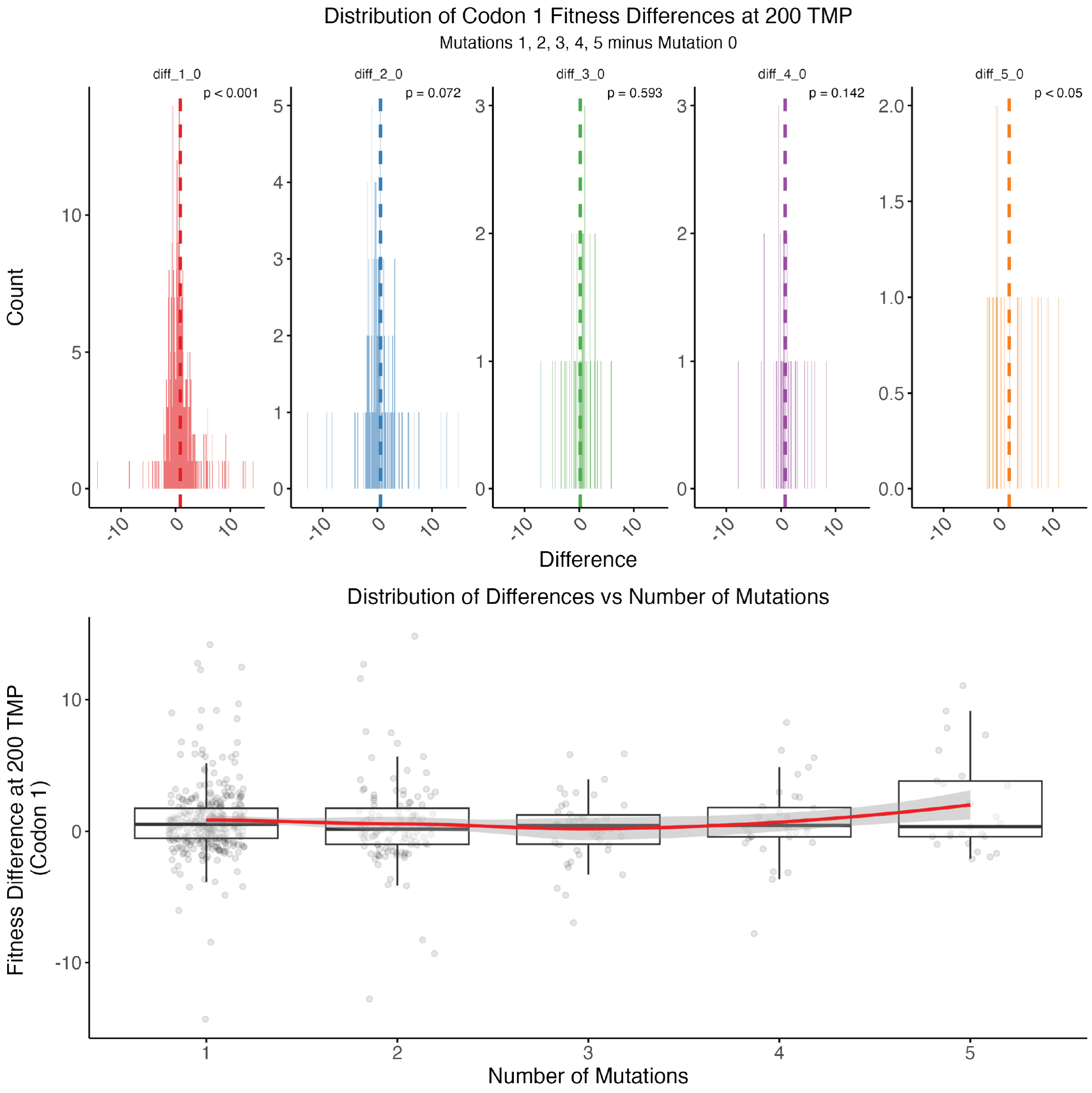

Fig. S9. Codon 1 Fitness Differences by Mutations at 200 µg/mL Trimethoprim. Distribution of mutant fitness responses at 200 µg/mL TMP for Codon 1, showing significant differences between single amino acid mutations and the perfect assemblies ($p < 0.001$), as well as for mutations with 5 amino acid differences compared to the perfect assemblies ($p < 0.05$). Mutants with 5 amino acid differences exhibit a significant positive impact on fitness at 200 µg/mL TMP.

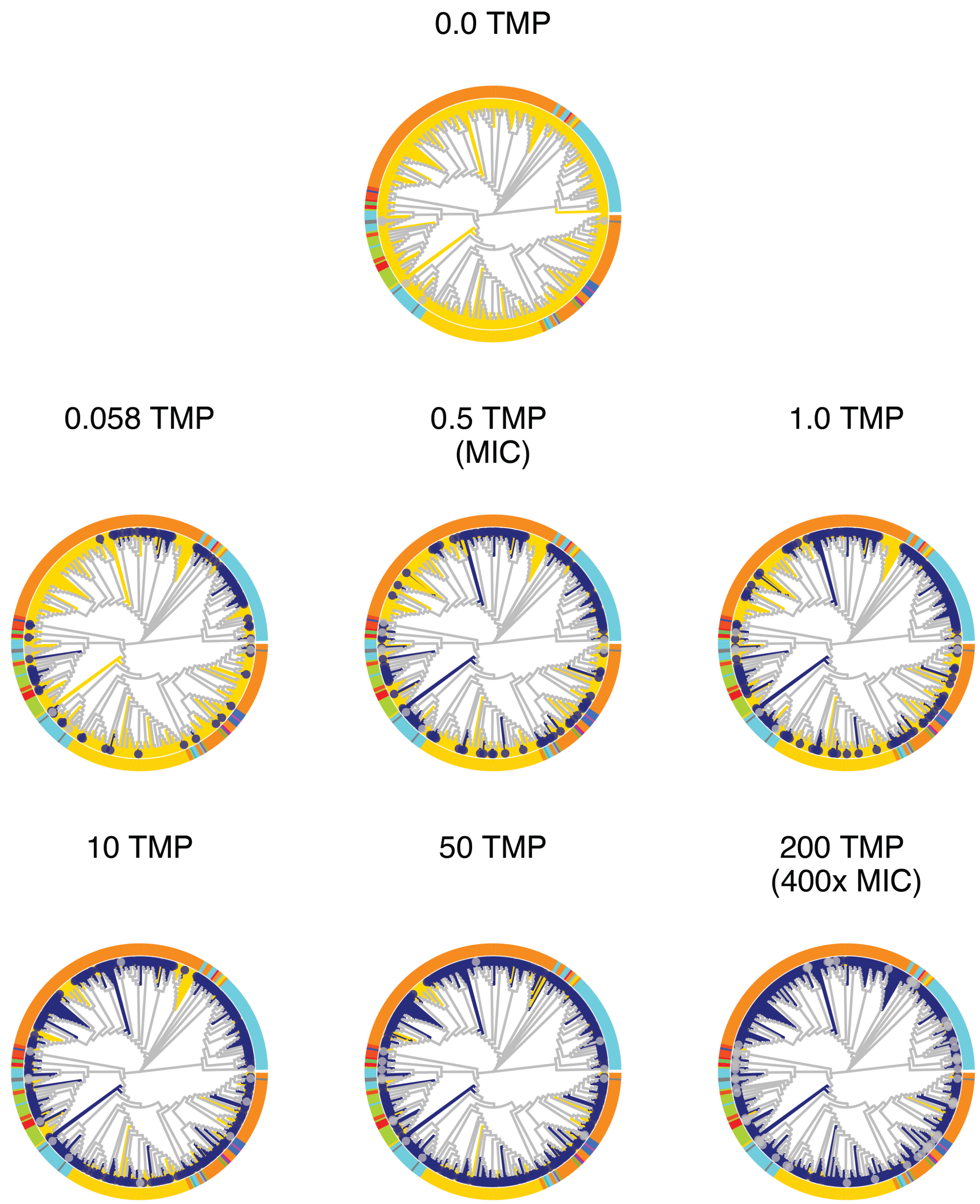

Fig. S10. Phylogenetic Fitness Distribution of DHFR Homologs Across Trimethoprim Gradient. Maximum likelihood phylogenetic trees of 416 DHFR homologs, showing trimethoprim-resistant homologs (fitness > -1) in yellow, and non-resistant homologs (fitness < -1) in blue across the trimethoprim (TMP) gradient.

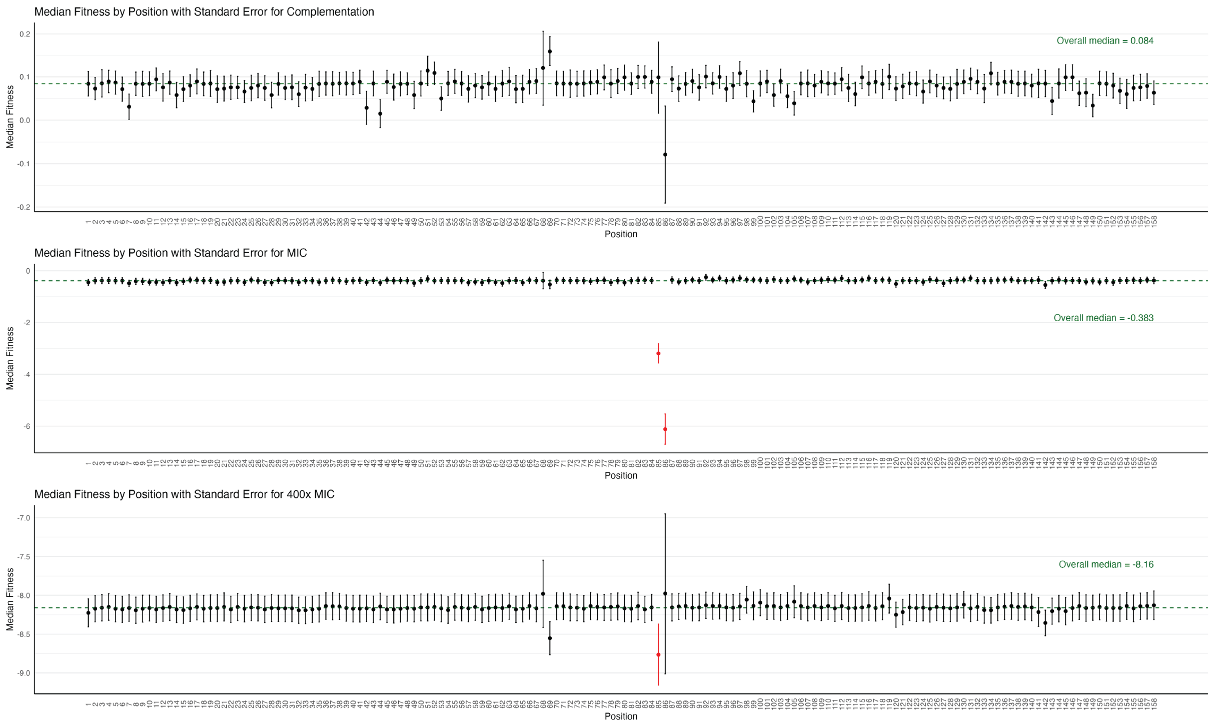

Fig. S11. Median Fitness by Position Across TMP Gradient. Points indicate the median fitness for recovered amino acids at each position under complementation (**top**), MIC (**middle**), and 400× MIC (**bottom**) conditions, with error bars representing the standard error of the median fitness. Red points highlight positions with median fitness significantly different from all others under those conditions. The green dashed line shows the overall median fitness across all positions.

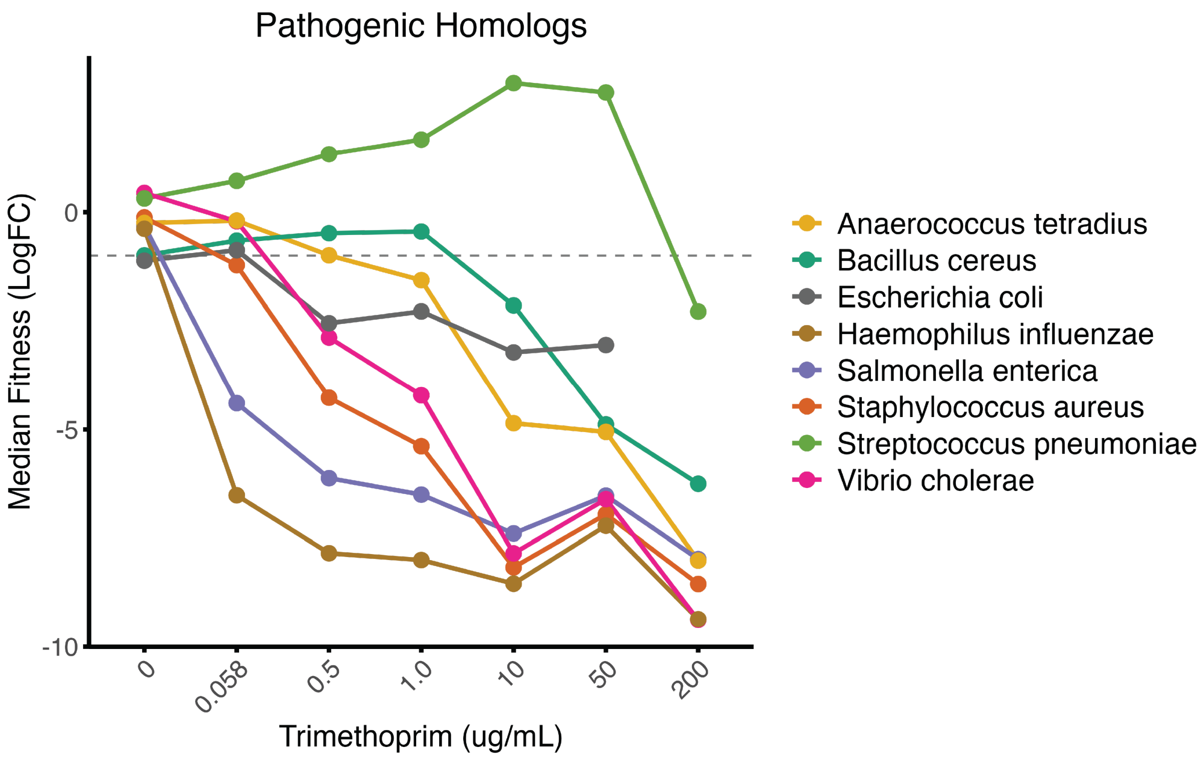

Fig. S12. Trimethoprim Fitness for Pathogenic DHFR Homologs. Median fitness for DHFR homologs across the trimethoprim gradient corresponding to the following pathogenic variants: *Anaerococcus tetradius*, *Bacillus cereus*, *Haemophilus influenzae*, *Salmonella enterica*, *Staphylococcus aureus*, *Streptococcus pneumoniae*, and *Vibrio cholerae*. The wild-type (WT) *E. coli* DHFR homolog is included as the positive control.

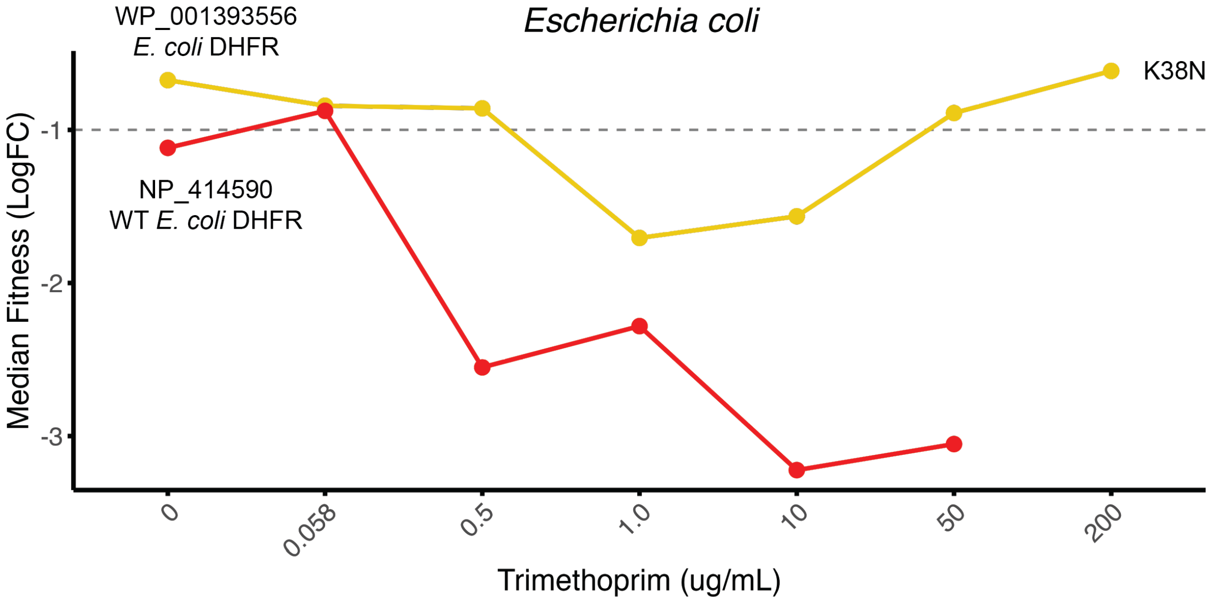

Fig. S13. Fitness of *E. coli* DHFR K38N Variant Relative to Wild-Type. Comparison of median fitness across the TMP concentration gradient between the wild-type *E. coli* DHFR (NP_414590) and a closely related variant (WP_001393556) containing a single amino acid substitution (K38N). This mutation confers increased resistance relative to the wild-type across all TMP concentrations.

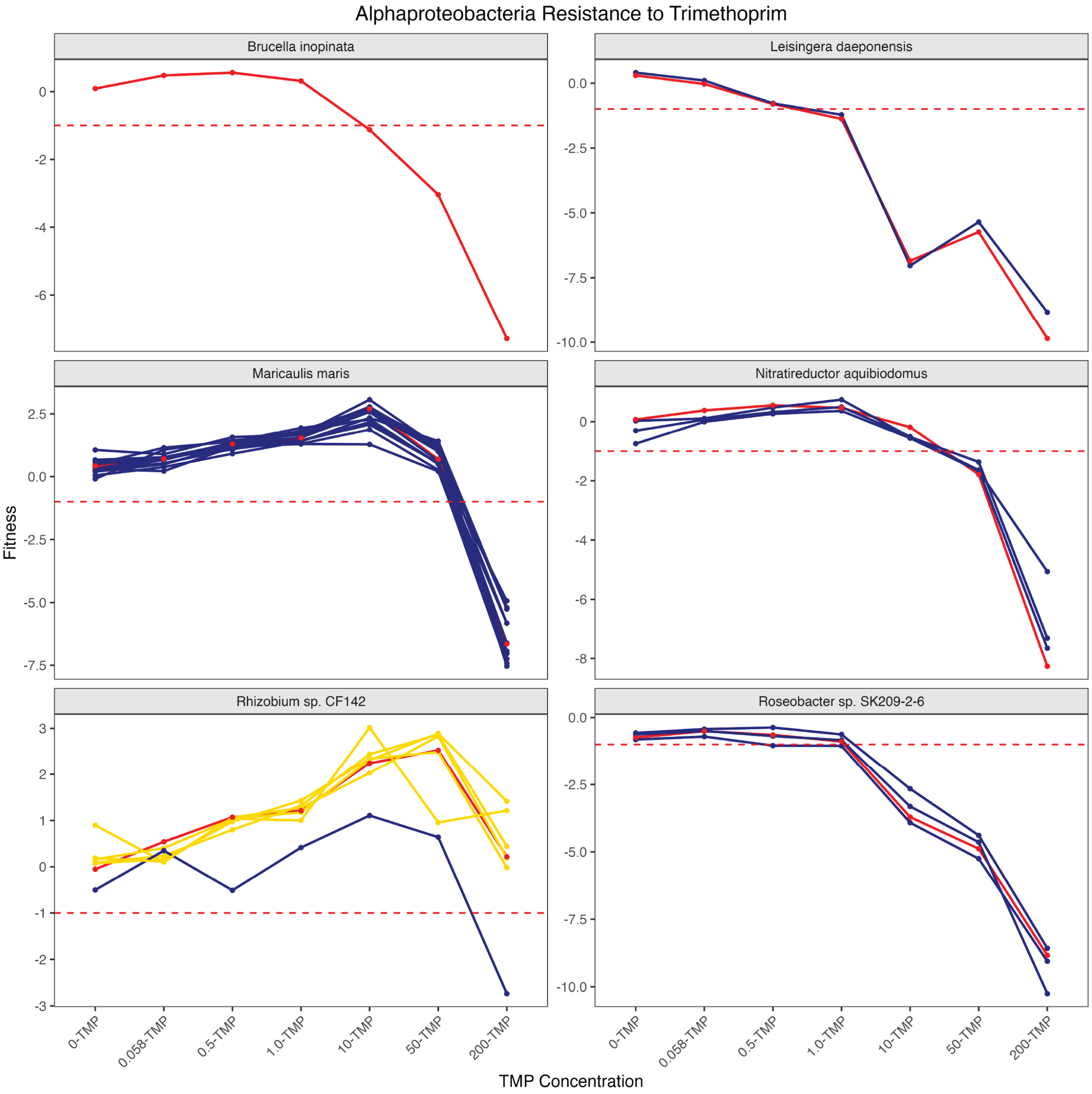

Fig. S14. Alphaproteobacteria Fitness. Median fitness (Log_2 fold-change) of six recovered Alphaproteobacteria species across the TMP gradient, showing the reference sequence in red, non-resistant mutants (fitness < -1) in blue, and TMP-resistant mutants (fitness > -1) in yellow. Only species with complete fitness data for the reference sequence and associated mutants were retained.

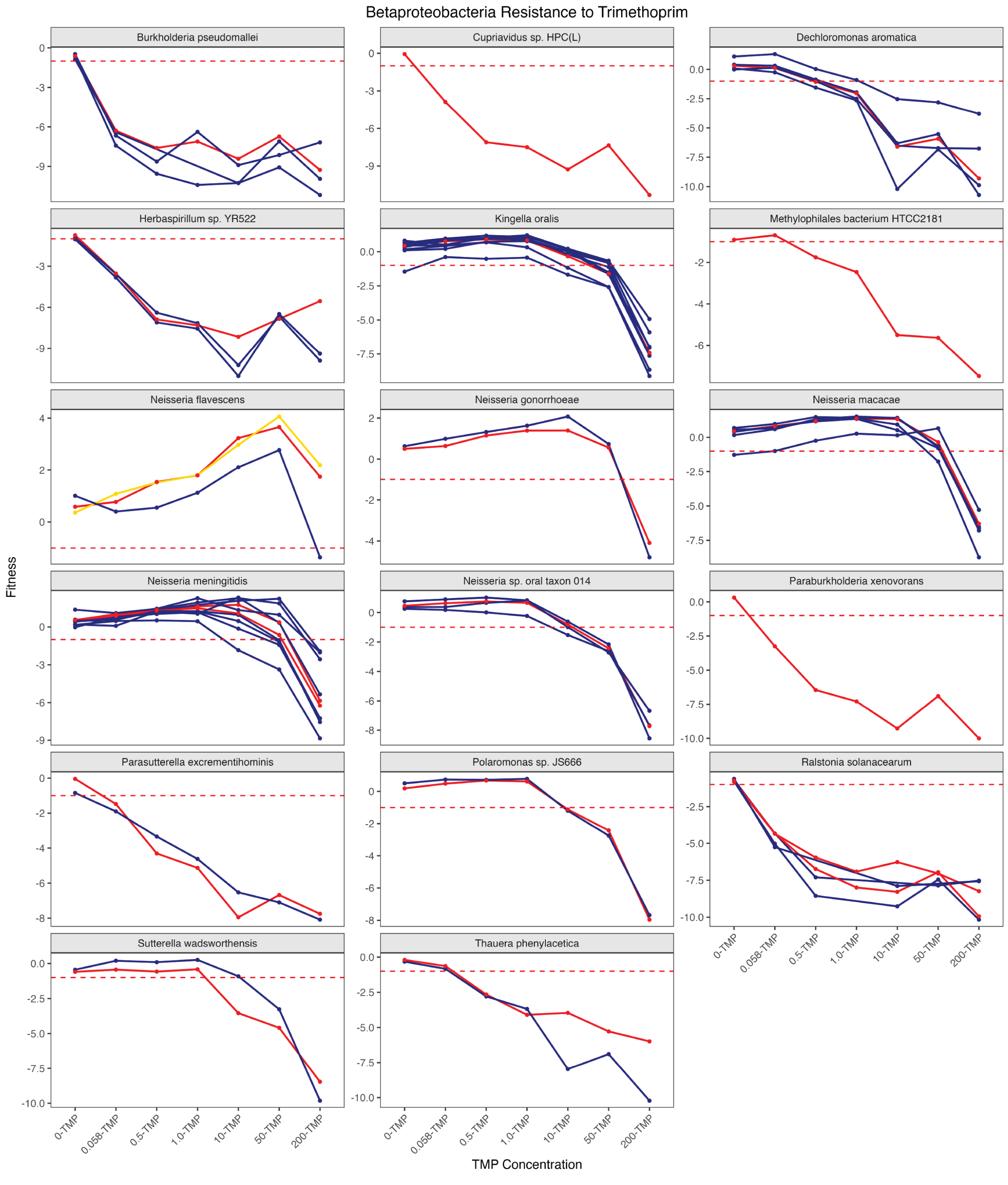

Fig. S15. Betaproteobacteria Fitness. Median fitness of 17 recovered Betaproteobacteria species across the TMP gradient, showing the reference sequence in red, non-resistant mutants (fitness < -1) in blue, and TMP-resistant mutants (fitness > -1) in yellow. Only species with complete fitness data for the reference sequence and associated mutants were retained.

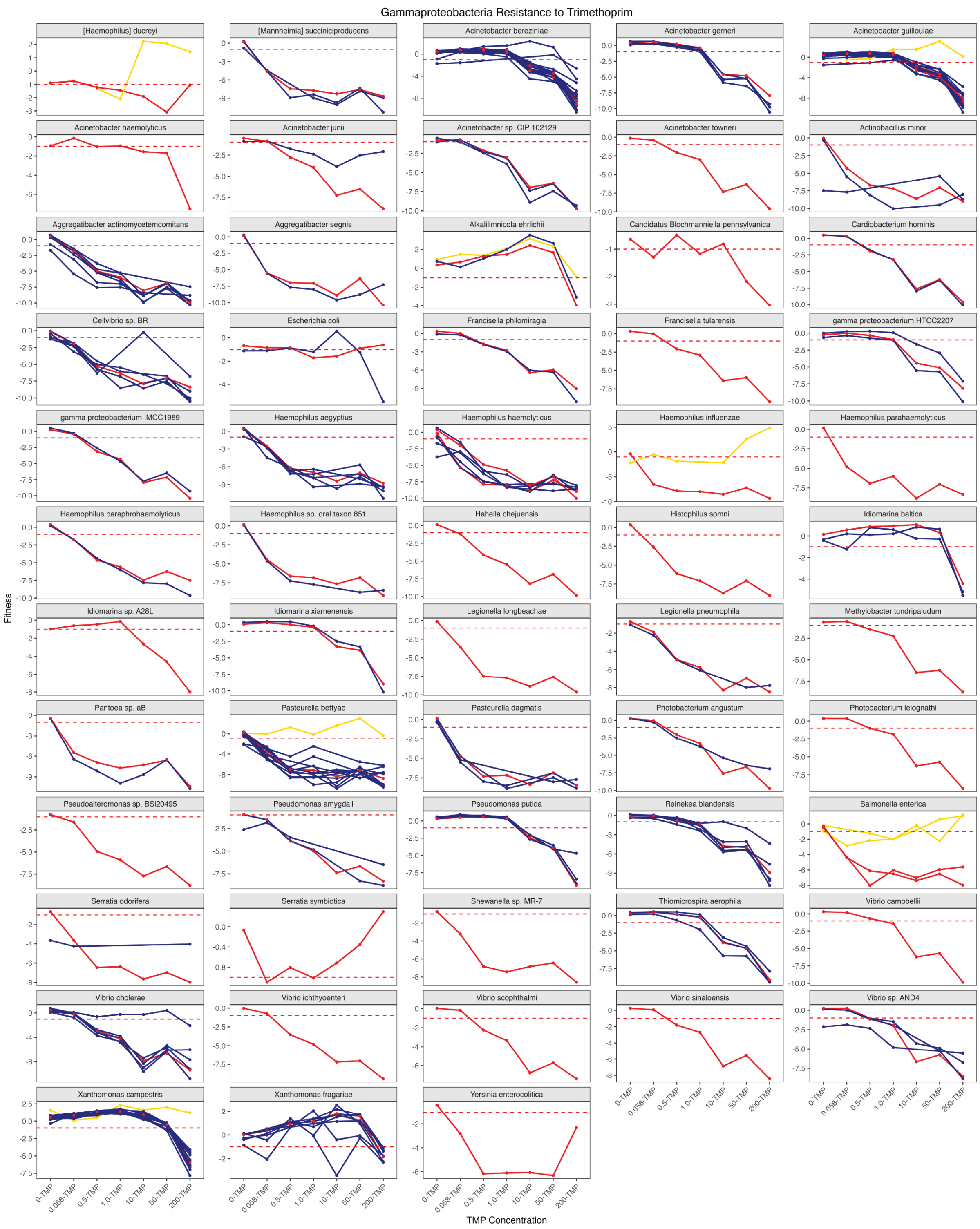

Fig. S16. Gammaproteobacteria Fitness. Median fitness of 58 recovered Gammaproteobacteria species across the TMP gradient, showing the reference sequence in red, non-resistant mutants (fitness < -1) in blue, and TMP-resistant mutants (fitness > -1) in yellow. Only species with complete fitness data for the reference sequence and associated mutants were retained.

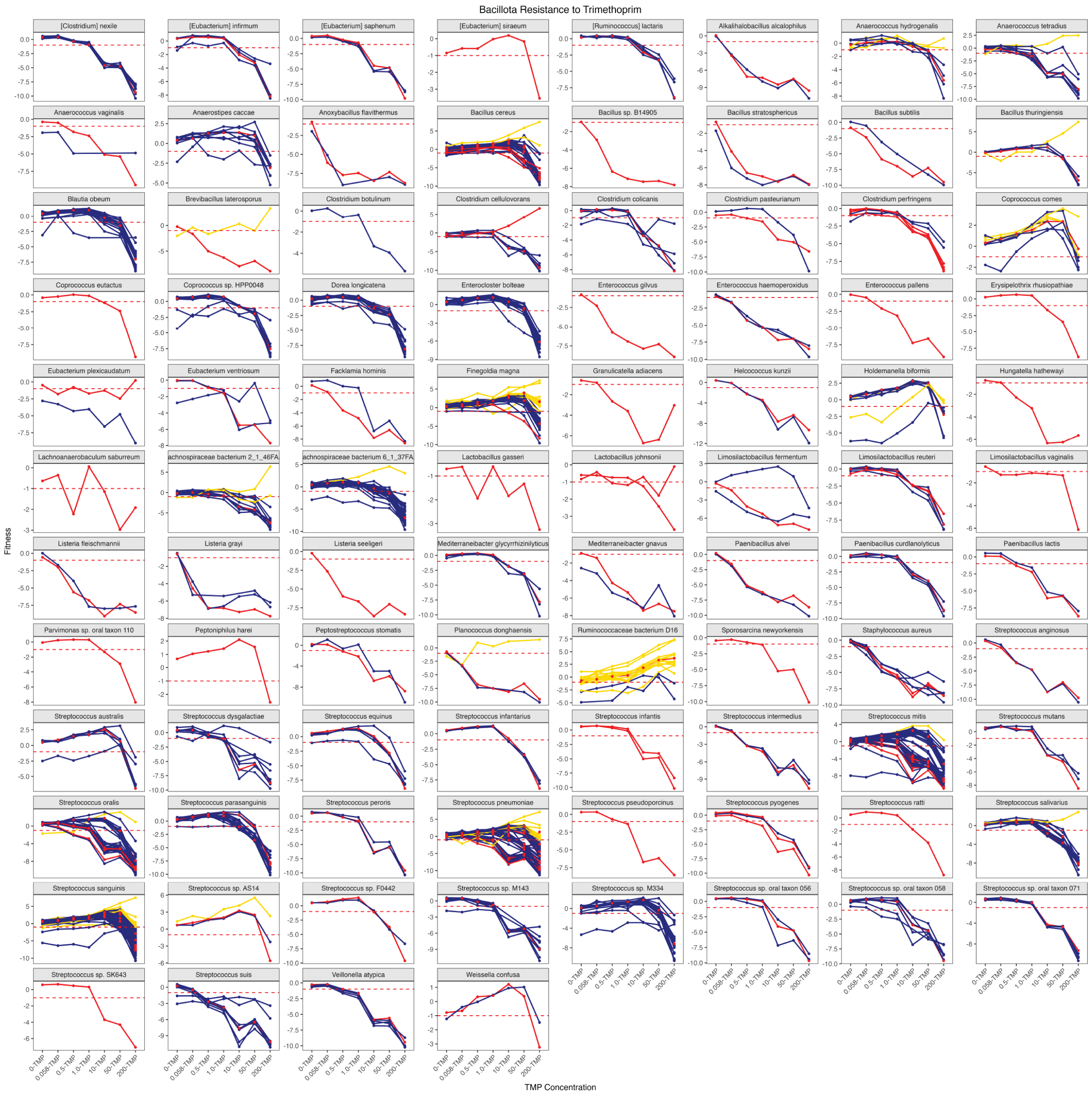

Fig. S17. Bacillota Fitness. Median fitness of 92 recovered Bacillota species across the TMP gradient, showing the reference sequence in red, non-resistant mutants (fitness < -1) in blue, and TMP-resistant mutants (fitness > -1) in yellow. Only species with complete fitness data for the reference sequence and associated mutants were retained.

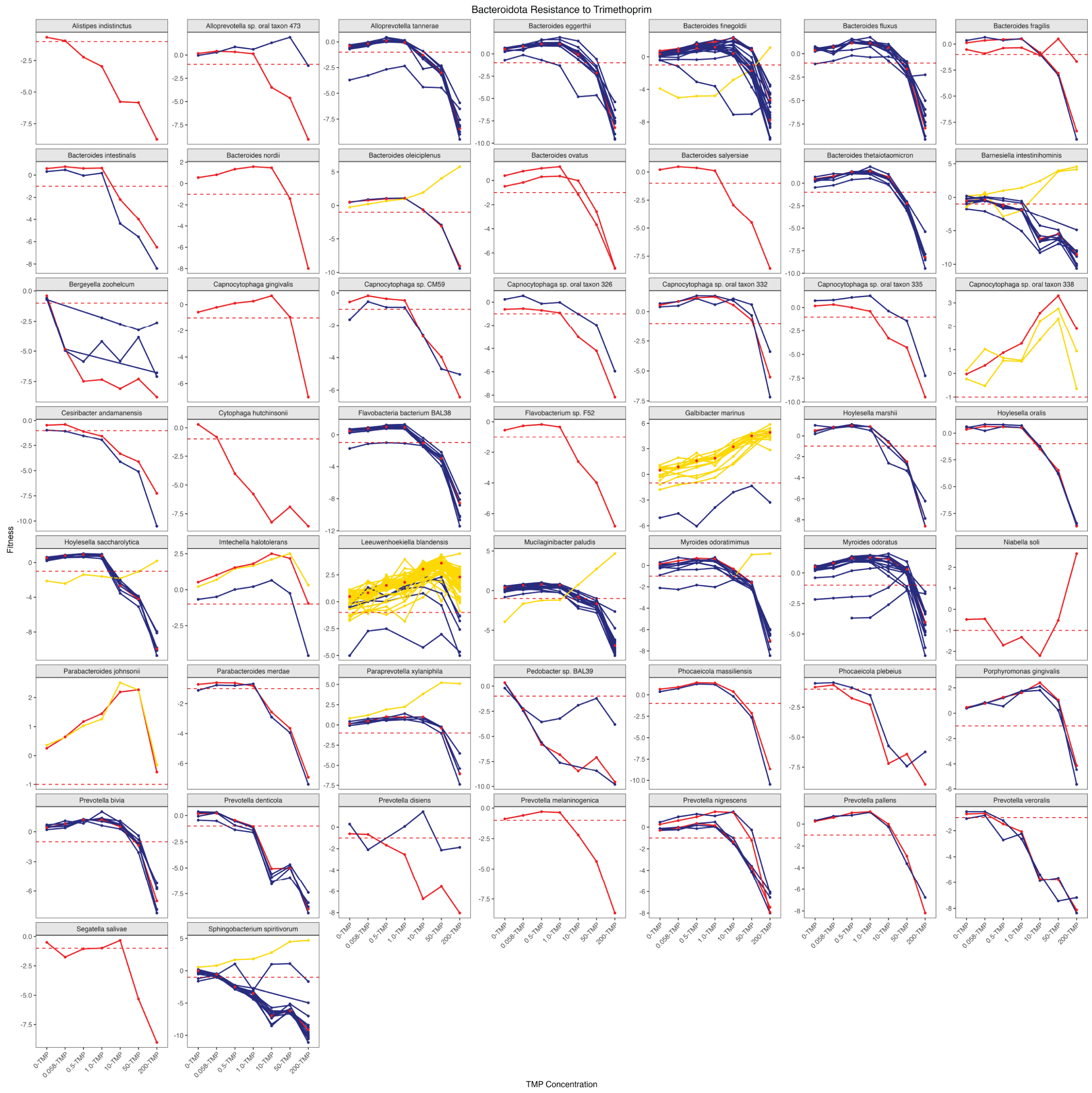

Fig. S18. Bacteroidota Fitness. Median fitness of 51 recovered Bacteroidota species across the TMP gradient, showing the reference sequence in red, non-resistant mutants (fitness < -1) in blue, and TMP-resistant mutants (fitness > -1) in yellow. Only species with complete fitness data for the reference sequence and associated mutants were retained.

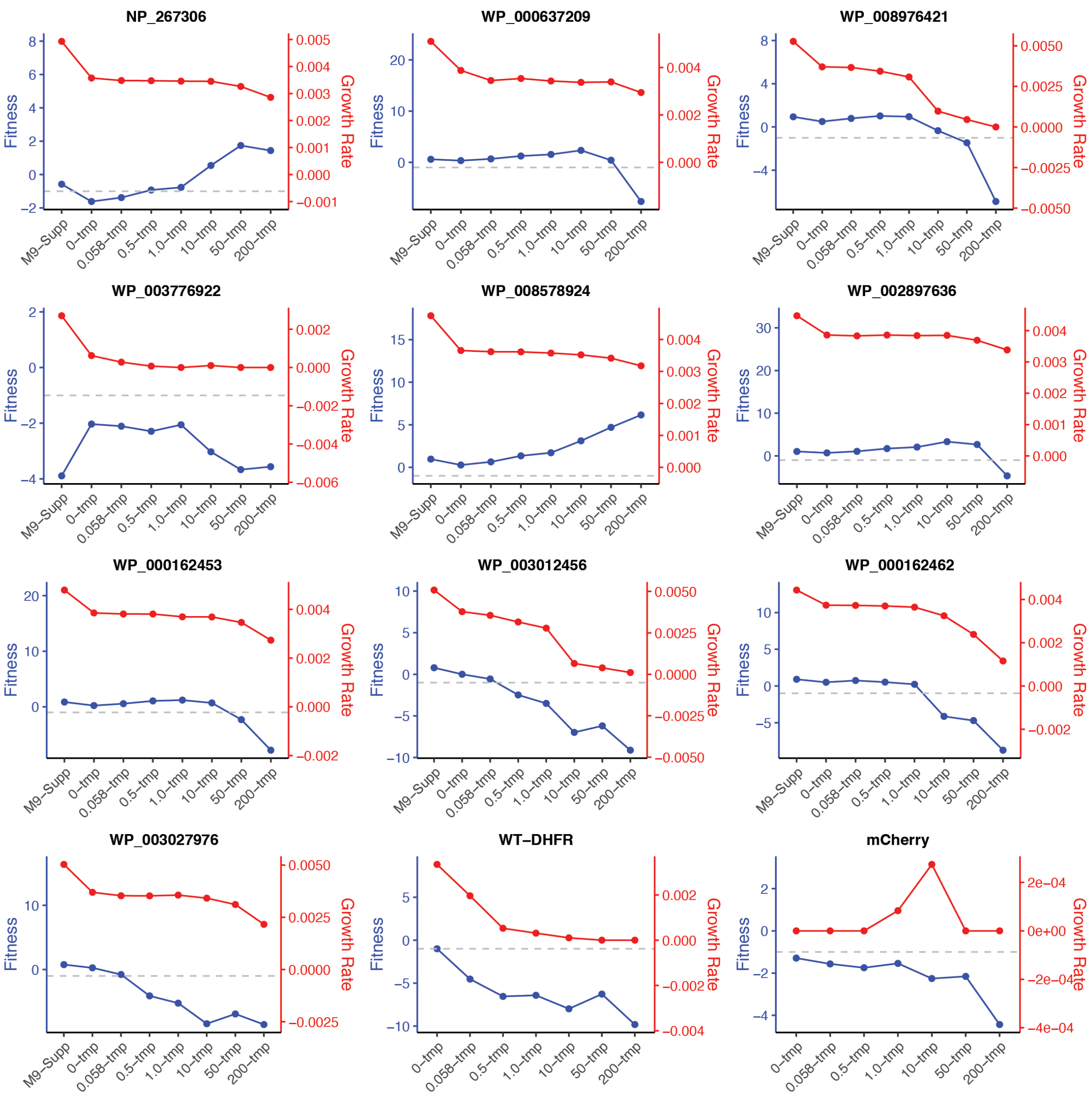

Fig. S19. Comparison of Dial-out Fitness and Growth Rates. Line plots showing experimentally determined fitness from pooled assays (blue) and growth rates of individual homologs measured via Dial-out PCR and plate reader assays (red). Growth rate (min^{-1}) is calculated as the maximum slope of OD600 vs. time on a log-linear plot. Plots represent 10 individual homologs spanning fitness values from strongly depleted (fitness < -9) to highly resistant (fitness > 6) under 200 µg/mL trimethoprim. Wild-type (WT) *E. coli* DHFR serves as the positive control, and mCherry as the negative control.

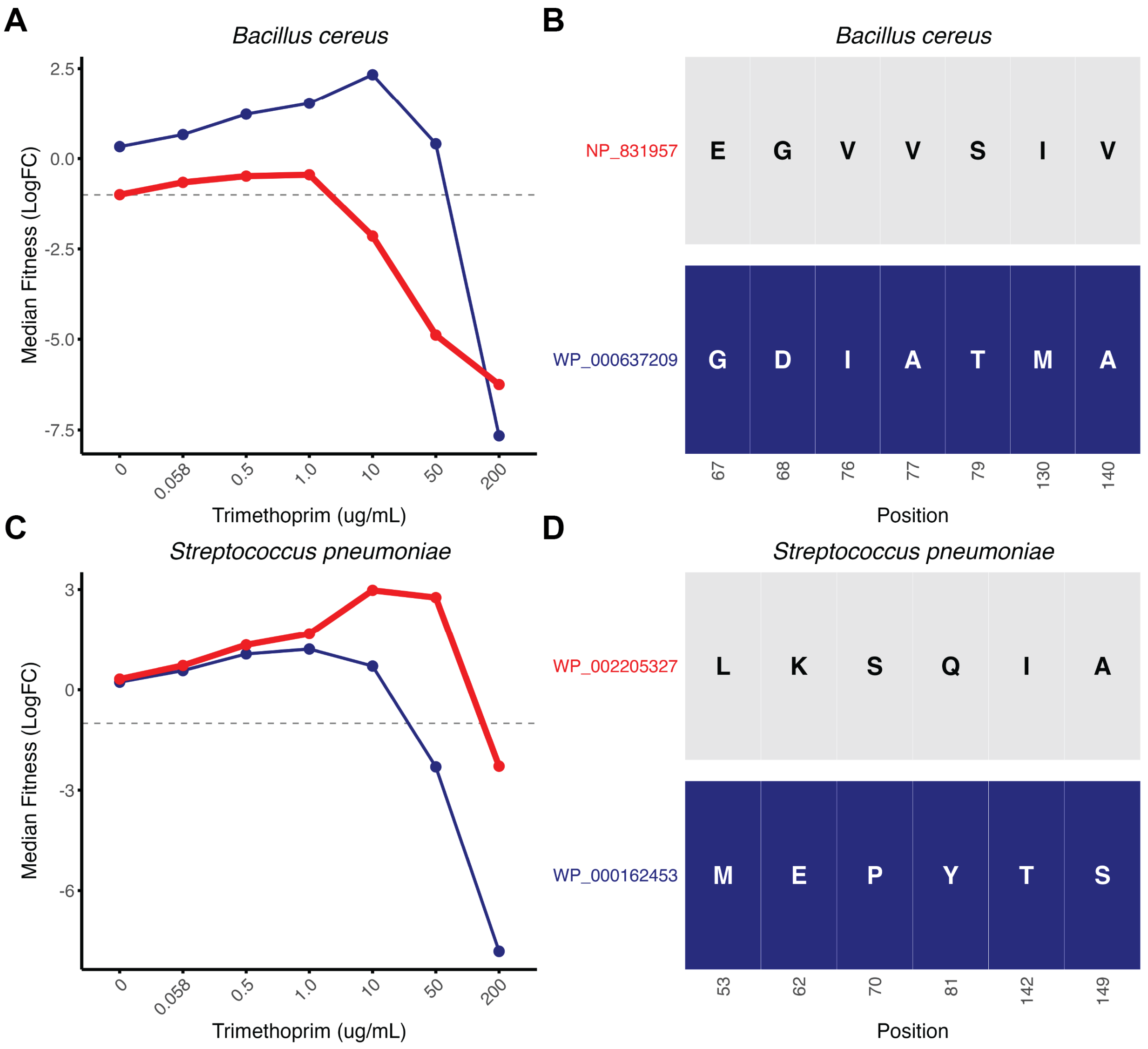

Fig. S20. Representative DHFR Homologs of Pathogenic Bacteria used as Dial-out Variants. (**A**) Median fitness of *Bacillus cereus* across the TMP gradient, comparing the reference variant (NP_831957; red line) for the resistance analysis with the dial-out variant (WP_000637209; blue line) used for fitness validation. (**B**) Grid plot of amino acid substitutions in *B. cereus* (NP_831957; top row) showing 7 unique residue substitutions in the dial-out variant (WP_000637209; bottom row). (**C**) Median fitness of *Streptococcus pneumoniae* across the TMP gradient, comparing the reference variant (WP_002205327; red line) for the resistance analysis with the dial-out variant (WP_000162453; blue line) used for fitness validation. (**D**) Grid plot of amino acid substitutions in *S. pneumoniae* (WP_002205327; top row) showing 6 unique residue substitutions in the dial-out variant (WP_000162453; bottom row).

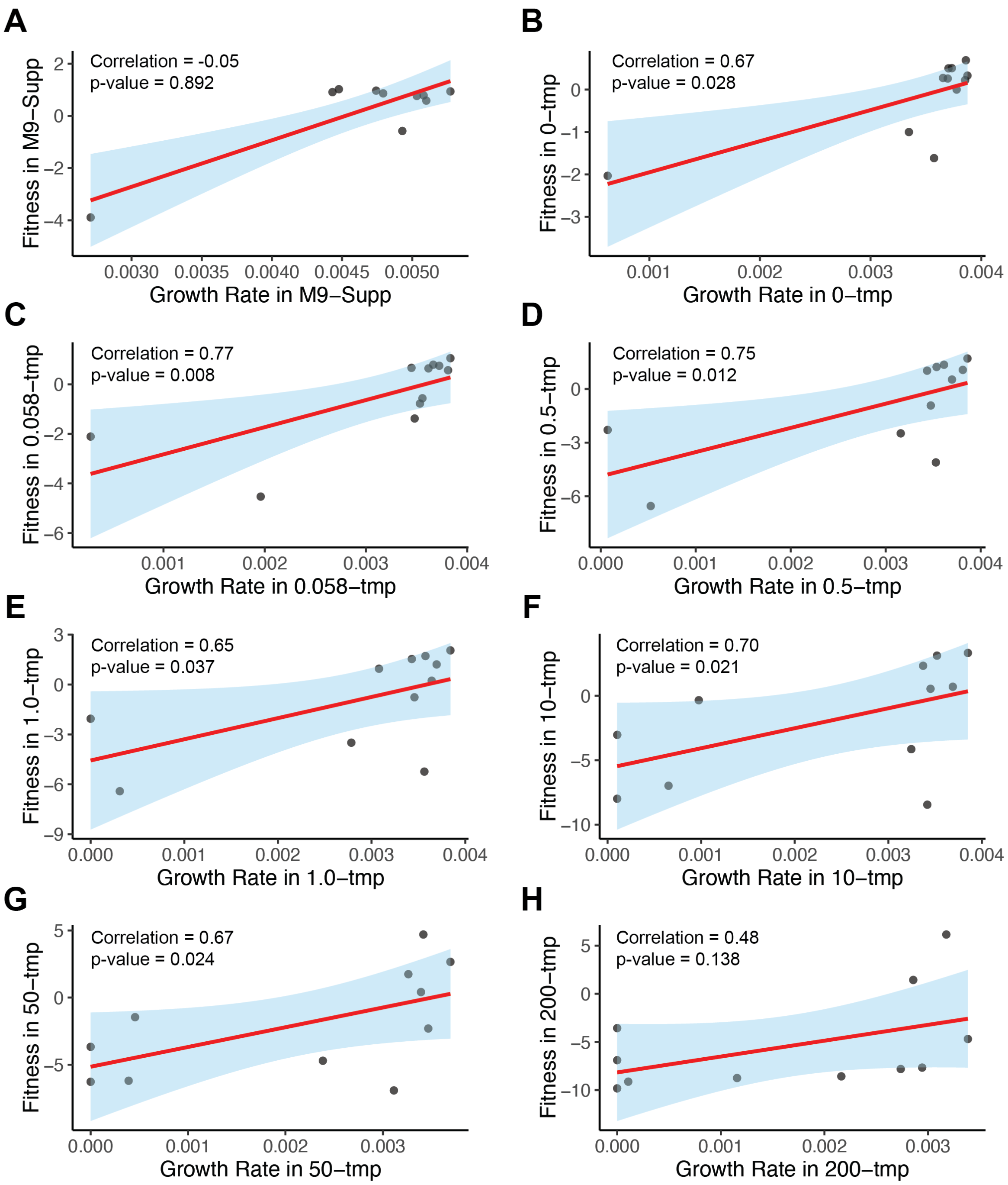

Fig. S21. Correlation Between Pooled Fitness and Dial-out Growth Rates. Growth rates of individual homologs, measured via dial-out PCR and plate reader assays, are compared to corresponding fitness values from pooled assays. Spearman's correlation (r_s, red line) with a 95% confidence interval (blue ribbon) is shown for: (**A**) M9-Supp media, (**B**) M9 media without TMP (Complementation), (**C**) M9 media with 0.058 µg/mL TMP, (**D**) M9 media with 0.5 µg/mL TMP (MIC), (**E**) M9 media with 1.0 µg/mL TMP, (**F**) M9 media with 10 µg/mL TMP, (**G**) M9 media with 50 µg/mL TMP, and (**H**) M9 media with 200 µg/mL TMP (400× MIC). Growth rate (min^{-1}) is defined as the maximum slope of OD600 vs. time on a log-linear plot. Points represent 10 individual homologs and the wild-type *E. coli* DHFR homolog (positive control) spanning fitness values from strongly depleted (median log_2 fold-change = -9.1) to highly resistant (median log_2 fold-change = +6.2) under 200 µg/mL trimethoprim.

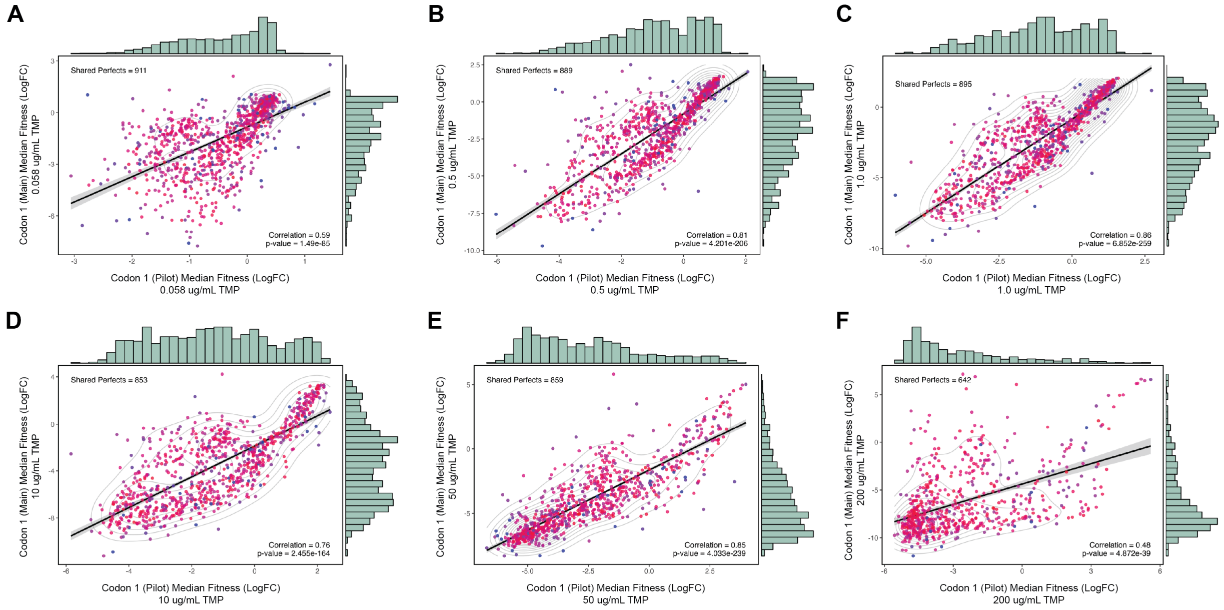

Fig. S22. Fitness Correlations for Homologs Shared Between Codon 1 Pilot and Main Dataset. Pearson correlations of fitness values for homologs shared between the Codon 1 pilot and main datasets at increasing trimethoprim (TMP) concentrations: (**A**) 0.058 µg/mL, (**B**) 0.5 µg/mL (MIC), (**C**) 1.0 µg/mL, (**D**) 10 µg/mL, (**E**) 50 µg/mL, and (**F**) 200 µg/mL (400× MIC). Each point represents a shared homolog, with point colors reflecting the number of recovered barcodes supporting each homolog in the datasets.

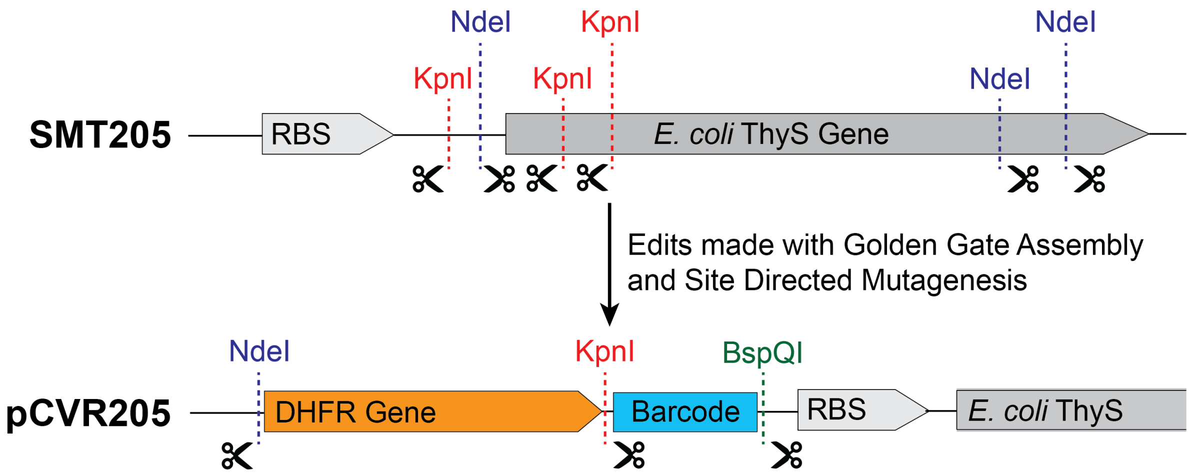

Fig. S23. pCVR205 Plasmid Construction. The DHFR expression vector for *E. coli* was derived from the SMT205 plasmid (Addgene #134817). SMT205 was modified to remove unnecessary restriction enzyme sites used for cloning the library. Three restriction sites were then reintroduced to align with the *folA* gene library. To generate the final plasmid, pCVR205, NdeI and KpnI cut sites were removed from the wild-type *thyA* gene, and a gBlock was used to eliminate restriction enzyme sites and insert one KpnI and one BspQI site at the 3' end of the DHFR gene's C-terminus. This was achieved via Golden Gate assembly, integrating the gBlock into the SMT205 backbone.

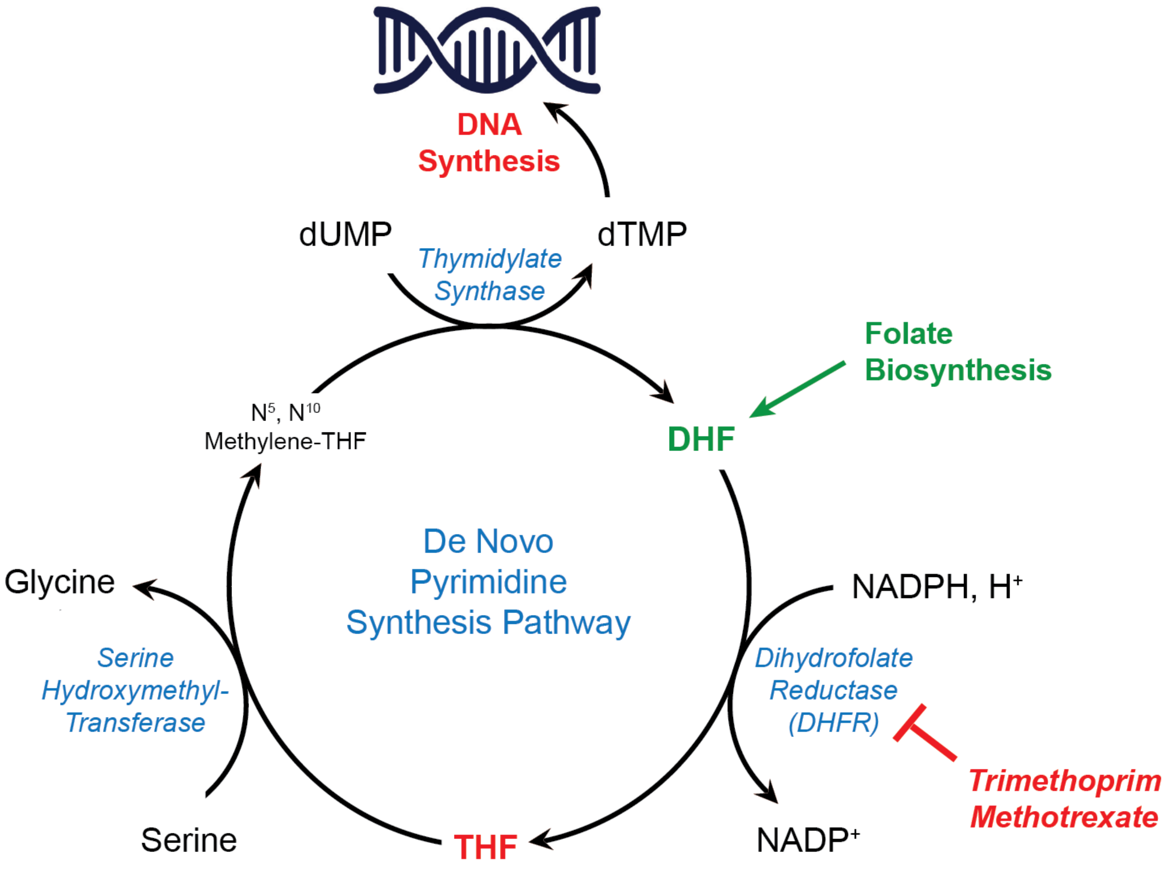

Fig. S24. Pyrimidine Synthesis. Dihydrofolate reductase (DHFR) catalyzes the reduction of dihydrofolate (DHF) to tetrahydrofolate (THF) with the cofactor NADPH. DHFR function is tightly coupled with thymidylate synthase. This cycle is an intermediate step in the synthesis of thymidine, one of the four canonical DNA bases. DHFR enzyme activity is inhibited by folate inhibitors such as trimethoprim and methotrexate.

Table S1. Phylogenetic Summary of DropSynth-Assembled DHFR Homologs. Phylogenetic summary of 1,208 designed DHFR homologs recovered from the original 1,536 DropSynth library.

Count	Taxonomy	Count	Domain	Count	%	Phylum
2	Domain	1,188	Bacteria	478	40.1	Pseudomonadota
19	Phylum	18	Archaea	472	39.6	Bacillota
31	Class	2	Virus	118	9.9	Bacteroidota
74	Order			61	5.1	Actinomycetota
159	Family			19	1.6	Mycoplasmatota
330	Genus			18	1.5	Euryarchaeota (Archaea)
778	Species			6	0.5	Chlamydiota
				3	0.3	Myxococcota
				3	0.3	Planctomycetota
				3	0.3	Spirochaetota
				3	0.3	Thermotogota
				2	0.2	Uroviricota (Virus)
				1	0.1	Bdellovibrionota
				1	0.1	Candidatus Saccharibacteria
				1	0.1	Cyanobacteria
				1	0.1	Fusobacteriota
				1	0.1	Lentisphaerota
				1	0.1	Thermodesulfobacteriota
				1	0.1	Verrucomicrobiota

Table S2. Sequence and Barcode Summary. Summary of total sequences and barcodes (BC) recovered and mapped across all nine experimental conditions for both codon version libraries.

Codon Version	Media Condition	Raw Sequence Count	Mapped Sequence Count	Mapped Sequences (%)	Raw Barcode Count	Mapped Barcode Count	Mapped Barcodes (%)
Codon 1	LB	83,770,481	61,036,972	73	332,234	197,558	59
Codon 1	M9: Supplemented	108,604,061	79,808,887	73	359,595	145,597	40
Codon 1	M9: Non-Supplemented (0 ug/mL TMP)	54,119,812	39,528,966	73	246,911	95,345	39
Codon 1	M9: Non-Supplemented (0.058 ug/mL TMP)	47,847,019	34,955,335	73	174,073	87,057	50
Codon 1	M9: Non-Supplemented (0.5 ug/mL TMP)	40,802,227	29,804,232	73	161,495	76,336	47
Codon 1	M9: Non-Supplemented (1.0 ug/mL TMP)	39,746,862	28,977,937	73	171,737	71,182	41
Codon 1	M9: Non-Supplemented (10 ug/mL TMP)	53,156,432	38,534,158	72	103,600	51,972	50
Codon 1	M9: Non-Supplemented (50 ug/mL TMP)	49,744,711	36,333,615	73	179,548	55,599	31
Codon 1	M9: Non-Supplemented (200 ug/mL TMP)	38,565,223	29,013,111	75	46,363	15,161	33
**Codon 1**	**Total Counts and Percentage Mapped**	**516,356,828**	**377,993,213**	**73**	**1,775,556**	**795,807**	**45**
Codon 2	LB	48,821,977	34,177,971	70	428,302	266,916	62
Codon 2	M9: Supplemented	65,871,632	45,234,084	69	409,052	191,349	47
Codon 2	M9: Non-Supplemented (0 ug/mL TMP)	68,405,404	47,717,303	70	291,387	141,835	49
Codon 2	M9: Non-Supplemented (0.058 ug/mL TMP)	43,914,011	30,754,811	70	215,066	126,987	59
Codon 2	M9: Non-Supplemented (0.5 ug/mL TMP)	43,752,442	30,367,898	69	190,020	108,523	57
Codon 2	M9: Non-Supplemented (1.0 ug/mL TMP)	49,596,583	34,229,832	69	181,809	101,164	56
Codon 2	M9: Non-Supplemented (10 ug/mL TMP)	58,144,156	38,659,435	66	151,536	76,505	50
Codon 2	M9: Non-Supplemented (50 ug/mL TMP)	52,530,782	33,688,875	64	134,374	67,666	50
Codon 2	M9: Non-Supplemented (200 ug/mL TMP)	63,525,568	39,388,556	62	170,613	66,503	39
**Codon 2**	**Total Counts and Percentage Mapped**	**494,562,555**	**334,218,765**	**68**	**2,172,159**	**1,147,448**	**53**
**Both**	**Total Counts and Percentage Mapped**	**1,010,919,383**	**712,211,978**	**70**	**3,947,715**	**1,943,255**	**49**

Table S3. Summary statistics for barcode counts. Summary statistics comparing raw barcode counts and high-confidence filtered barcodes for designed DHFR homologs (0 mutations) and mutant variants with up to five amino acid substitutions, across both codon-optimized library versions. Only barcodes supported by ≥10 sequencing reads were retained, and mutant variants were required to be detected across the full TMP selection gradient. This filtering ensures barcode-level replication and reliable interpretation of fitness measurements.

Statistical Metric	Codon 1		Codon 2	
	Raw BCs	Filtered BCs	Raw BCs	Filtered BCs
Designed Homologs (0 mutations)				
Median	227	39	214	46
Mean	434	76	467	131
STD	537	99	631	204
Max	4311	823	4027	1709
Mutant Variants (1-5 mutations)				
Median	1	1	1	1
Mean	2.1	1.1	2.2	1.2
STD	2.9	0.4	4.6	0.9
Max	78	16	342	43

Table S4. Homolog Summary by Codon Library. Summary of unique homologs recovered from the DropSynth-assembled mapping effort, raw sequence counts derived from the complementation assay conditions (min. 1 sequence per barcode), filtered sequence counts derived from the complementation assay conditions (min. 10 sequences per barcode), and filtered sequence counts with a min. 5 unique barcodes per homolog in the complementation assay conditions.

Codon Version	Mapping Homologs (DropSynth)	Raw Count Homologs (min. 1 seq)	Filtered Count Homologs (min. 10 seqs)	Filtered BC Homologs (min. 5 BCs)
**Codon 1**	1,048	973	961	797
**Codon 2**	904	840	818	666
**Shared**	744	663	643	493
**Unique**	464	487	493	477
**Total**	1,208	1,150	1,136	970

Table S5. False-positive Rates. False-positive rates (fitness ≥ -1) were estimated using highly mutated variants (>50 amino acid substitutions) with a minimum of 5 unique barcodes.

Experimental Condition	Codon 1			Codon 2		
	Total Mutants (mutations > 49)	False-Positive Mutants (fitness > -1)	False-Positive Rate (%)	Total Mutants (mutations > 49)	False-Positive Mutants (fitness > -1)	False-Positive Rate (%)
Complementation	16,239	96	0.6%	15,121	197	1.3%
MIC (TMP)	12,160	81	0.7%	11,321	159	1.4%
400x MIC (TMP)	1,068	13	1.2%	5,222	30	0.6%

Table S6. Complementation of Archaeal and Viral DHFR Homolog Outgroups. Summary of the fitness outcomes of 8 archaeal and 2 viral DHFR homologs included as phylogenetic outgroups in the DropSynth-assembled library. These sequences were intentionally incorporated to evaluate the functional limits of DHFR complementation in *E. coli* and to explore whether DHFR variants from evolutionarily distant sources can support folate metabolism in a bacterial host. Fitness values are reported across the TMP gradient, with variants capable of complementation (fitness ≥ -1) shown in **bold**.

mutID	Domain	Phylum	Source	Similarity to *E. coli* (%)	0 TMP (ug/mL)	0.058 TMP (ug/mL)	0.5 TMP (ug/mL)	1.0 TMP (ug/mL)	10 TMP (ug/mL)	50 TMP (ug/mL)	200 TMP (ug/mL)
**WP_005576873**	**Archaea**	**Euryarchaeota**	**Natronobacterium gregoryi**	**47.4**	**0.00**	**-0.85**	**NA**	**NA**	**-1.72**	**NA**	**NA**
**WP_007976376**	**Archaea**	**Euryarchaeota**	**Haladaptatus paucihalophilus**	**52.8**	**-0.14**	**0.01**	**0.08**	**-0.48**	**0.17**	**-1.68**	**NA**
WP_004043660	Archaea	Euryarchaeota	Haloferax volcanii	44.4	-1.59	-1.78	0.46	-1.80	NA	-2.58	NA
WP_004057195	Archaea	Euryarchaeota	Haloferax mediterranei	47.2	-1.05	-1.44	-2.04	-2.10	-1.91	-1.73	-1.86
WP_004968047	Archaea	Euryarchaeota	Haloferax denitrificans	44.7	-1.85	-2.35	-1.83	NA	NA	NA	NA
WP_005555536	Archaea	Euryarchaeota	Natronococcus amylolyticus	47.7	-1.01	0.08	0.00	-2.14	NA	NA	NA
WP_007190543	Archaea	Euryarchaeota	Haloarcula californiae	47.3	-3.90	-4.22	-4.32	-4.34	-5.29	-4.92	-6.46
WP_007994849	Archaea	Euryarchaeota	Halorubrum arcis	45.1	-1.19	-0.62	0.34	-0.83	-4.03	-1.84	NA
**NP_944205**	**Viruses**	**dsDNA viruses**	**Aeromonas phage Aeh1 (Aeh1)**	**36.6**	**0.11**	**-0.05**	**-0.99**	**-1.63**	**-6.09**	**-5.93**	**-7.10**
NP_803570	Viruses	dsDNA viruses	Pseudomonas phage phiKZ	39.3	-2.75	-2.64	-3.45	-4.13	-5.76	-5.67	-10.31

Table S7. Resistant Homologs Recovered from 200 ug/mL TMP Condition. Summary table of the top 16 homologs with sufficient fitness (> -1) to retain function under the highest TMP assay condition. Codon indicates sufficient fitness (> -1) in Codon 1, Codon 2, or both.

Codon	mutID	fitD11D03	fitE06D04	Phylum	Species
Codon 1	WP_003686654	1.74	-3.64	Betaproteobacteria	*Neisseria flavescens*
Codon 1	WP_002839715	1.6	-5.3	Bacillota	*Finegoldia magna*
Codon 1	WP_003498667	0.92	-3.23	Bacillota	*NA*
Codon 1	WP_008152545	-0.58	-5.39	Bacteroidota	*Parabacteroides johnsonii*
Codon 1	WP_004442738	-0.6	-6.28	Alphaproteobacteria	*NA*
Codon 1	WP_008237079	-0.96	-3.81	Bacteroidota	*Imtechella halotolerans*
Codon 2	WP_008754633	-1.91	-0.59	Bacillota	*Lachnoanaerobaculum saburreum*
Codon 2	WP_008664019	-8.5	3.75	Bacteroidota	*NA*
Codon 2	WP_009161670	-9.93	2.08	Bacteroidota	*Hoylesella saccharolytica*
Both	WP_010075211	6.62	5.48	Bacillota	*Clostridium cellulovorans*
Both	WP_007654866	6.48	5.41	Bacteroidota	*NA*
Both	WP_008990832	4.94	1.96	Bacteroidota	*Galbibacter marinus*
Both	WP_008583481	2.65	1.78	Bacteroidota	*Niabella soli*
Both	WP_009778768	2.3	1.48	Bacteroidota	*Leeuwenhoekiella blandensis*
Both	NP_229441	-0.55	-0.77	Thermotogota	*Thermotoga maritima*
Both	WP_008390643	-0.71	0.21	Bacillota	*NA*

Table S8. Resistant Homolog Summary Across TMP Gradient. Summary of the number of perfect assembly homologs resistant to trimethoprim (fitness > -1) across the TMP gradient. The total homolog count varies between TMP treatments based on recovery rates.

Homolog Fitness	0 ug/mL TMP	0.058 ug/mL TMP	0.5 ug/mL TMP	1.0 ug/mL TMP	10 ug/mL TMP	50 ug/mL TMP	200 ug/mL TMP
Complement (fit > -1)	416	318	246	226	128	80	27
Dropout (fit < -1)	0	92	162	183	276	321	332
Total Homologs	416	410	408	409	404	401	359

Table S9. Phylogenetic Fitness Differences Across TMP Gradient. Summary statistics for absolute differences in fitness between homolog pairs based on evolutionary distance for each TMP treatment. Close-related pairs have an absolute difference in evolutionary distance < 1, whereas far-related pairs have an absolute difference in evolutionary distance > 1. Fitness differences are calculated as the absolute value of the difference between fitness values of two taxa, resulting in a unitless quantity.

Treatment	Group	N	Mean	SD	Min	Max
0.058	Close	5,814	0.70	0.79	0	5.87
	Far	161,876	1.50	1.51	0	9.05
0.5 (MIC)	Close	5,812	1.48	1.40	3.44E-04	8.65
	Far	160,244	2.71	2.32	6.28E-06	10.08
1	Close	5,812	1.88	1.70	3.06E-04	9.29
	Far	161,060	3.09	2.48	1.67E-05	10.22
10	Close	5,744	3.68	3.19	2.23E-03	12.79
	Far	157,068	4.31	3.04	0	13.69
50	Close	5,764	2.88	2.57	3.21E-04	11.42
	Far	154,636	3.34	2.62	2.12E-05	13.40
200 (400x MIC)	Close	5,254	2.24	2.40	2.60E-04	15.54
	Far	123,268	3.14	3.43	0	18.20

Table S10. Dial-out Variant Pooled Fitness Summary. Median fitness for pooled DHFR dial-out variants across the trimethoprim (TMP) gradient.

mutID	M9-Supp	0-TMP	0.058-TMP	0.5-TMP	1.0-TMP	10-TMP	50-TMP	200-TMP
NP_267306	-0.58	-1.62	-1.38	-0.93	-0.77	0.55	1.74	1.44
WP_000637209	0.58	0.33	0.66	1.23	1.53	2.33	0.41	-7.66
WP_008976421	0.94	0.50	0.79	1.02	0.95	-0.34	-1.46	-6.89
WP_003776922	-3.89	-2.03	-2.11	-2.29	-2.06	-3.03	-3.67	-3.56
WP_008578924	0.97	0.27	0.64	1.35	1.72	3.12	4.70	6.16
WP_002897636	1.02	0.69	1.05	1.70	2.05	3.33	2.66	-4.69
WP_000162453	0.86	0.22	0.57	1.06	1.21	0.70	-2.31	-7.81
WP_003012456	0.79	0.00	-0.56	-2.48	-3.50	-6.98	-6.20	-9.12
WP_000162462	0.91	0.51	0.75	0.53	0.23	-4.14	-4.71	-8.74
WP_003027976	0.76	0.26	-0.78	-4.11	-5.24	-8.45	-6.92	-8.57
WT-DHFR	NA	-1.00	-4.53	-6.54	-6.42	-7.99	-6.27	-9.82
mCherry	NA	-1.29	-1.57	-1.75	-1.54	-2.26	-2.16	-4.44

Table S11. Dial-out Variant Taxonomic Summary. Phylogenetic taxonomic classification for each dial-out variant and percent sequence identity to *E. coli*. IDs in **bold** correspond to the pathogenic homologs *Bacillus cereus* and *Streptococcus pneumoniae*.

mutID	Sequence Identity to E. coli (%)	Phylum	Class	Order	Family	Genus	Species
NP_267306	45.2	Bacillota	Bacilli	Lactobacillales	Streptococcaceae	Lactococcus	Lactococcus lactis
**WP_000637209**	**45.7**	**Bacillota**	**Bacilli**	**Bacillales**	**Bacillaceae**	**Bacillus**	**Bacillus cereus**
WP_008976421	45.1	Bacillota	Clostridia	Lachnospirales	Lachnospiraceae	*NA*	Lachnospiraceae bacterium
WP_003776922	44.5	Bacillota	Bacilli	Lactobacillales	Carnobacteriaceae	Alloiococcus	Alloiococcus otitis
WP_008578924	38.1	C. Saccharibacteria	*NA*	*NA*	*NA*	*NA*	candidate division TM7 genomosp. GTL1
WP_002897636	44.1	Bacillota	Bacilli	Lactobacillales	Streptococcaceae	Streptococcus	Streptococcus sanguinis
**WP_000162453**	**44.0**	**Bacillota**	**Bacilli**	**Lactobacillales**	**Streptococcaceae**	**Streptococcus**	**Streptococcus pneumoniae**
WP_003012456	50.6	Bacteroidota	Sphingobacteriia	Sphingobacteriales	Sphingobacteriaceae	Sphingobacterium	Sphingobacterium spiritivorum
WP_000162462	44.0	Bacillota	Bacilli	Lactobacillales	Streptococcaceae	Streptococcus	Streptococcus mitis
WP_003027976	41.8	Bacillota	Bacilli	Lactobacillales	Streptococcaceae	Streptococcus	*NA*

Table S12. Primers for Plasmid Preparation. Primer pairs for plasmid preparation and gBlock sequence designed to insert one KpnI site and one BspQI site at the 3′ end of the DHFR gene. This gBlock was integrated into the SMT205 backbone using Golden Gate assembly, creating the plasmid pCVR1.

Primer Pair Name	Forward Sequence (5’-->3’)	Reverse Sequence (5’-->3’)
pCVR205_SDM	tatgatcagtctgattgcggcgttagcggtagatcgcgttaTCGGCATGGAAAACGCCA	tgatatctcattattaaagttaaacaaaattatttctacaggGGAATTGTTATCCGCTCACAATTC
SMT205_bb	TGACCAGGTCTCGGCAATTGAGCCGTGAGCCG	TGACCAGGTCTCGCCAGAATCTCAAAGCAATAGCTGTGA
mi3_R1	GTGGAATTGTGAGCGGATAACAATTTCACACAGGAAACAGCTCATATG	*NA*
EV4_BspQI	*NA*	cagtctaGCTCTTCatgaCGCACATTTCCCCGAAAAGTGCCACCTGACGTCg

**gBlock Sequence from IDT**
TGACCAGGTCTCGCTGGAGCGGCGGTAAGGTACCTAAGTGTGGCTGCGGAACGCA<br>CGACGTCAGGTGGCACTTTTCGGGGAAATGTGCGTCATGAAGAGCCAGGCGTCGAC<br>AAGCTTGCGGCCGCATAATGCTTAAGTCGAACAGAAAGTAATCGTATTGTACATCCC<br>TATCAGTGATAGAGATTGACATCCCTATCAGTGATAGAGATACTGAGCACATCAGCA<br>GGACGCACTGACCGAATTCATTAAAGAGGAGAAAGGGACCACATGGCAGATCTCAT<br>GAAACAGTACCTGGAGCTGATGCAAAAAGTTCTGGATGAGGGGACCCAGAAAAACG<br>ACCGCACGGGGACCGGAACGCTGAGCATTTTTGGCCATCAGATGCGCTTTAACCTG<br>CAGGACGGATTCCCGCTGGTTACGACCAAACGCTGCCACCTGCGTAGCATTATTCA<br>TGAGCTGCTGTGGTTTCTGCAAGGTGACACTAACATCGCGTATCTGCACGAAAACAA<br>TGTGACGATCTGGGATGAATGGGCCGATGAAAACGGCGATCTGGGCCCAGTGTATG<br>GTAAACAGTGGCGCGCCTGGCCAACGCCAGATGGCCGCCACATCGATCAGATCAC<br>GACCGTGCTGAATCAACTGAAAAACGACCCGGACAGCCGCCGCATTATTGTTTCCG<br>CGTGGAATGTGGGTGAACTGGATAAAATGGCGCTGGCGCCGTGCCATGCATTCTTC<br>CAGTTTTACGTTGCGGACGGTAAGCTGAGCTGTCAACTTTATCAGCGCAGCTGCGAT<br>GTTTTCCTCGGCCTGCCGTTCAATATCGCCAGCTACGCGTTACTGGTGCACATGATG<br>GCGCAGCAGTGCGACCTGGAGGTGGGTGATTTTGTCTGGACCGGCGGTGATACCCA<br>CCTGTACAGCAACCACATGGACCAAACGCATCTGCAACGAGACCTCGTCA

Table S13. iTag Illumina Prep Primers. PCR was performed to amplify plasmid barcodes using five primer pairs that introduced custom sequencing adapters and library indexes compatible with Illumina sequencing.

Primer Pair Name	Forward Sequence (5'-->3')	Reverse Sequence (5'-->3')
CVR205stubBC1_FWD	GCTCTTCCGATCTNGGTACCtaaGTGTGGCTGCGGAAC	GCTCTTCCGATCTNGTGCCACCTGACGTCgtgc
CVR205stubBC2_FWD	GCTCTTCCGATCTNNGGTACCtaaGTGTGGCTGCGGAAC	GCTCTTCCGATCTNNGTGCCACCTGACGTCgtgc
CVR205stubBC3_FWD	GCTCTTCCGATCTNNNGGTACCtaaGTGTGGCTGCGGAAC	GCTCTTCCGATCTNNNGTGCCACCTGACGTCgtgc
CVR205stubBC4_FWD	GCTCTTCCGATCTNNNNGGTACCtaaGTGTGGCTGCGGAAC	GCTCTTCCGATCTNNNNGTGCCACCTGACGTCgtgc
CVR205stubBC5_FWD	GCTCTTCCGATCTNNNNNGGTACCtaaGTGTGGCTGCGGAAC	GCTCTTCCGATCTNNNNNGTGCCACCTGACGTCgtgc

Table S14. Dial-out PCR Primers. Dial-out PCR primers were designed to flank each homolog construct, with reverse primers annealing to the gene-specific barcode.

mutID	Primer Pair	Barcode	Forward Sequence (5'-->3')	Reverse Sequence (5'-->3')
NP_267306	NP_06_FWD<br>NP_06_REV	ACCCGGCGTGGATATATCTA	GATATCATATGATCAT<br>TGGCATCTGGG	TAGATATATCCACG<br>CCGGGT
WP_000637209	WP_09_FWD<br>WP_09_REV	AAACCGAATTTTTGCATGGA	GATATCATATGATCGT<br>GAGCTTTATGGTT	GCTCCATGCAAAAA<br>TTCGGTTT
WP_008976421	WP_21_FWD<br>WP_21_REV	AAAGCGTCGTGTAAGCGATC	GATATCATATGAACAT<br>TATTGTCGCGGTTG	GATCGCTTACACGA<br>CGCTTT
WP_003776922	WP_22_FWD<br>WP_22_REV	ACTCGACCTCTAAAAATCTT	GATATCATATGATTGC<br>CTACGTTTGG	GCAAGATTTTTAGA<br>GGTCGAGT
WP_008578924	WP_24_FWD<br>WP_24_REV	TACACTGCCCTGAATTATCT	GATATCATATGAAAGT<br>GTTTCTGATTGTCGC	GCAGATAATTCAGG<br>GCAGTGTA
WP_002897636	WP_36_FWD<br>WP_36_REV	CCAGTAGGAGCAAAGTCTAC	GATATCATATGACGAA<br>AAAAATCATTGCA	GTAGACTTTGCTCC<br>TACTGG
WP_000162453	WP_53_FWD<br>WP_53_REV	CATCTTAGTCCTCGATTTAT	GATATCATATGACCAA<br>AAAGATTGTCGC	GTGCATAAATCGAG<br>GACTAAGATG
WP_003012456	WP_56_FWD<br>WP_56_REV	ATCGCACGGGTCTGCGCCGA	GATATCATATGTCGCA<br>CCTGAAAATCAC	CATACACGCACTAC<br>CCCTGT
WP_000162462	WP_62_FWD<br>WP_62_REV	AGTGGGCATTAGTATAGATT	GATATCATATGACTAA<br>AAAAATTGTGGCG	GCAATCTATACTAAT<br>GCCCACT
WP_003027976	WP_76_FWD<br>WP_76_REV	CAACTTAGTTTTGAATTGGA	GATATCATATGATTAA<br>AAAAATTGTGGCGAT	GTGCTCCAATTCAA<br>AACTAAGTTG
